# Sequential boundary tracking via reinforcement learning for overlapping cell resolution in sickle cell imaging

**DOI:** 10.1038/s41598-026-67069-w

**Published:** 2026-08-21

**Authors:** Saira Batool, Min Guo, Muhammad Nabeel Asghar, Sajid Iqbal

**Affiliations:** 1 Information and Communication Engineering, North University of China, Taiyuan, 030051 China; 2 College of Economics and Management, North University of China, Taiyuan, China; 3 Department of Information Systems, College of Computer Sciences and Information Technology, King Faisal University, Al-Ahsa, Saudi Arabia

**Keywords:** Hybrid model, Reinforcement learning, Q-learning, Feature extraction, Red blood cell classification, Computational biology and bioinformatics, Engineering, Health care, Mathematics and computing

## Abstract

Sickle cell disease requires rapid, accurate diagnostic screening to facilitate early therapeutic actions with potential life-saving impact. Unfortunately, traditional manual microscopy screening is heavily affected by high rates of human error, long processing delays, and detection variability, which are especially pronounced when identifying truly challenging microscopic fields of view, such as those consisting of clusters of overlapping red blood cells. These diagnostic delays result in poor patient outcomes, particularly in resource-limited settings where specialized healthcare providers are scarce. Highly precise automated tracking solutions are thus essential to ensure diagnostic fairness and reliability. To address these challenges, we introduce a general end-to-end hybrid in which feature extraction is based on deep localized features and decision-making is performed by sequential RL. The method is based on a dual-stage pipeline: a bespoke U-Net architecture first separates individual cells within complex, overlapping cell bunches, and afterward, a Q-learning agent that finds optimized sequential tracking paths on the coordinate grid matrices maps the cells to final labels. Extensive experimental results on the public Kaggle dataset show that the segmentation module achieves a high-fidelity dice similarity coefficient of 95.51%, and the proposed integrated RL pathfinder improves the final classification results, achieving an overall accuracy of 98.00%. Our solution achieves a +16:60% absolute diagnostic improvement over a traditional single CNN classifier (81:40%), demonstrating that the proposed framework is robust, has novel methodological characteristics, and is clinically viable for fully automated hematological screening.

## Introduction

Sickle cell disease can shorten life, damage many organ systems, hurt kidney function, and cause permanent disability. People with SCD may have times when they feel fatigued, ill, and yellow. It can also cause serious problems, including damage to the eyes, heart, lungs, liver, kidneys, and joints^1,2^. Sickle cell disease (SCD) is an inherited condition caused by genetic mutations. It causes acute chest syndrome and gallbladder disease, even in kids^3^. It affects hemoglobin, the main component of red blood cells^4,5^. When the parents are transporters, there is a 25% chance that the teens will also become transporters, a 25% chance that they will not be affected, and a 50% chance that they will be affected^6–8^. America is the most affected area, with many people suffering from SCD^9^. One in every 16,300 babies born in Hispanic America has SCD^10^. In North America, mortality rates from complications associated with SCD have been significantly reduced due to the implementation of extensive blood testing and newborn screening techniques. To manage SCD, diagnostic tools must be more accurate and easier to access^11,12^.

Different image processing and machine learning techniques^13,14^ have been used to classify, measure, and separate sickle cell anemia in red blood cells. These methods work well, but they are sensitive to changes in cell size, color, and shape. Moreover, the effectiveness of these strategies depends on the chosen attributes. The authors of a study^15^ proposed a simple yet effective SVM-based method for detecting acute lymphoblastic leukemia, achieving 96% accuracy. The authors in^16^ suggested a random forest-based model for classifying AML. The paper^17^ presents an effective AML detection framework that employs MobileNetV2. The importance of deep learning (DL) in medical imaging is growing, driven by the availability of many different tools and methods, as well as new digital medical technologies. When used for complex image analysis tasks such as Sickle Cell Anemia (SCA) segmentation and classification, traditional machine learning (ML) methods often face problems that deep learning methods don’t^18,19^. Traditional machine learning methods require extensive and often arduous manual feature engineering^20,21^.

As image datasets grow larger and more complex, traditional machine learning methods often struggle to perform well. As image resolution increases or cell morphology becomes more detailed, it becomes increasingly difficult to create durable, hand-crafted features. The features derived from conventional machine learning are often low-level in nature^22^. These algorithms struggle to capture the hierarchical and abstract representations needed to distinguish subtle variations in cell morphology, such as the different phases or types of sickled cells^23^. Deep learning has overcome the challenges mentioned above and demonstrated remarkable efficacy across a diverse array of tasks. Deep learning can independently extract features and execute classification in a single step^24^. This research presents a hybrid methodology for classifying SCA into two distinct categories: normal and sickle cells. Data augmentation has resolved the issue^25,26^. The proposed models accurately and efficiently differentiate between the two types of sickle cells, thereby aiding in assessing the severity of sickle cell anemia^27^. Although both U-Net and classical Q-learning are well-established methods, combining them into an interactive, closed-loop spatial pathfinder that models overlapping red blood cell classification as a penalty-based MDP navigation problem is a modern technique. The motivation for developing the proposed automated diagnostic tool stems from permanent clinical requirements and stark computational constraints in existing state-of-the-art pipelines for analysis of medical images:

- *Healthcare necessity and disparity* Sickle cell disease (SCD) remains a major burden on healthcare systems worldwide, with early pediatric screening essential to prevent lethal vaso-occlusive crises. Specialized hematologists are virtually nonexistent in the developing world or rural regions. Rigid microscopic smear analysis is physically demanding, tedious, and highly susceptible to observer subjectivity. A fully automated, robust tool is thus strongly motivated by the desire to make advanced diagnostic screening accessible to all.
- *Susceptibility to error propagation* as the segmentation stage is completely isolated from subsequent classification, errors achieved by single cells at boundary segmentation will directly propagate into the classifier, producing false negatives. When traditional models are faced with clumped, highly deformed, or overlapping red blood cells, errors in cell boundary segmentation propagate into the classifier, leading to alarming rates of false-negative diagnoses.

This work is directly inspired by the intuition that an interactive reinforcement learning agent, which can explore a pre-segmented pixel coordinate space through dynamic state-action feedback loops, can circumvent the brittleness often encountered with decoupled pipelines. Treating cell tracking as an intelligent pathfinding problem, our method effectively disambiguates pairs of neighboring cells, thereby recovering trust in automated, real-world diagnostics. A thorough examination of the state-of-the-art literature shows a clear gap in research on automated analysis of hematological images. While state-of-the-art Convolutional Neural Networks (CNNs) are well-suited to localized feature extraction, existing approaches for Sickle Cell Disease detection are afflicted by a conceptual bottleneck: they are constructed as separate, feed-forward pipelines. In the classic ones, an image is segmented, and the resulting static segment is blindly pushed to a downstream classifier. When dealing with complex blood smears in which normal erythrocytes and sickle cells are tightly clustered or overlap, the original segmentation boundaries become indistinct. Since the architecture is a strictly feed-forward network, segmentation errors propagate unchecked into the classification stage, leading to high false-negative and misclassification rates. There is a notable absence of adaptive, closed-loop schemes capable of traversing and self-correcting within dense cellular matrices. To address the gap, this paper proposes a unified dual-stage hybrid architecture that combines high-fidelity structural segmentation with a reinforcement learning (RL) decision-guidance agent. Rather than viewing classification as a single-step prediction performed on isolated boxes, we model the diagnostic pipeline as an interactive path-routing problem in an MDP environment. To clearly show the methodological novelty of this work, the main contributions of our hybrid RL-CNN framework are summarized as follows:

- *Novel closed-loop architectural fusion* We introduce a general approach that fuses a U-Net segmentation layer with an interactive Q-learning agent, breaking free from the usual reliance on brittle, decoupled sequential pipelines.
- *Dynamic state-space path routing* This study views cell classification as an active pathfinding rather than a static, one-shot box prediction. The agent treats the deep CNN feature maps as a set of localized environmental states and dynamically moves its tracking window across the coordinate grids to disambiguate cells.
- *Algorithmic path regularization via penalty-driven MDP* We propose analytically tractable mathematical reward function landscape under a temporal bellman optimality framework. This algorithm operates as an explicit spatial regularizer, through localized negative feedback loops to the boundary overruns and recurrent trajectories, the agent dynamically routes the paths out of ambiguous gray-zone in coordinate matrices, it is mathematically neutralized cascade errors rather than parameter tuning.
- *Validated empirical results* This study shows state-of-the-art results on public blood smear datasets, achieving a 95.51% dice coefficient in segmentation and 98.00% final classification accuracy, which is a +16.60% absolute gain in comparison to conventional standalone CNN models.

### Organization

This paper is structured as follows: Section "Related work" provides a comprehensive background analysis. Section "Proposed model" outlines the research methodology employed in this study. Section "Results and discussion" shows the results and talks about them. Section "Conclusion" summarizes the most important results of the study.

## Related work

Automated identification and discrimination of abnormal erythrocytes, particularly in sickle cell disease (SCD), are evolving rapidly from classical morphological image processing to deep learning methodologies. This part summarizes recent progress in deep learning methods, RL applications in healthcare, and the bottlenecks encountered in multi-stage constructs.

### Traditional classification methods

The authors of a study^3^ discovered that the traditional approach for identifying blood abnormalities is arduous and insufficiently precise. The authors use MATLAB’s image processing tools to convert blood smear images to black-and-white and binary formats. The method accurately counts leukocytes, erythrocytes, and sickle-shaped cells. After 40 difficult trials and a thorough study, the system demonstrated it was very good at what it did. Of the 40 trials, 25 found the right answer. However, this investigation included numerous trials and did not yield reliable data for sickle cell classification. In a separate study^28^, the authors contend that there is an imperative for a more expedited method of sickle cell classification that exceeds human analysis. Visual cues such as shape, color, and texture affect the features of the separated cytoplasm and nucleus. The derived characteristics are analyzed to determine the probable classification of blood cells as normal or pathological. In another study^29^, the authors employed Canny edge detection as a multi-stage technique to identify edges in images^30^, subsequently using those edges to extract features and categorize sickle cells as normal or sickle. This study employed advanced artificial intelligence techniques, including machine learning and deep learning, to develop a robust, effective model that addresses the challenges of traditional sickle cell classification methods.

### Machine learning approaches

Artificial imaging methodologies^31^ have demonstrated significant potential for analyzing medical images, particularly for recognizing and classifying sickle cells. The authors examine the application of machine learning methods to classify medical data, particularly for directing disease-modifying therapies in sickle cell disease. The study’s findings from different models show that recurrent networks didn’t perform as well as feedforward neural networks and the Random Forest model. The case study concentrates on the classification of medication dosage for individuals with sickle cell disease^32,33^. In a separate study^34,35^, the authors proposed a new hybrid segmentation method for distinguishing normal from sickle red blood cells in microscopic blood smears. This method aims to improve the accuracy and effectiveness of sickle cell anemia classification while automating detection. The hybrid methodology integrates fuzzy C-means and adaptive thresholding with four distinct thresholding techniques. The present study employed a machine-learning-driven reinforcement-learning methodology, specifically a Q-learning agent, to develop a hybrid model for the segmentation and classification of sickle cells.

### Deep learning in erythrocyte analysis and segmentation

In recent years, deep learning methods^36^ have become a powerful tool across many applications. For effective treatment and management of the condition, it is important to accurately and promptly identify and categorize anomalies. Most of the existing material is biased toward using machine learning to group and categorize red blood cells and sickle cells. The study^37,38^ sought to identify the most effective classification attributes and methodologies for diagnosing SCD. The study^39^ characterized and classified White Blood Cells (WBC). While training the CNN, ensemble architectures were tested using both full learning and transfer learning methods. At the end of the study, the system achieved 93% accuracy in identifying WBCs. ResNet50^40^, an architecture for convolutional neural networks, has shown better results in transfer learning. The authors utilized geostatistical modeling to ascertain the prevalence of SCD among Brazilian adults in the study^41^. As stated in^42^, the only way to find out a person’s blood type is to measure, calculate, and look at their skin.

The architectural limitations of standard pipelines persist, though deep learning-based methods have recently brought significant improvements to automated hematological analysis. For example, Jan et al.^43^ leveraged custom deep networks to perform semantic segmentation to separate leukemia-affected white blood cells from high background noise, but their method is still limited to segmentation rather than multi-class classification. Tackling multi-class pipelines, Mondal et al.^44^, stacked a basic U-Net segmentation layer with a lightweight CNN to classify nine different blood cell categories. Yet, like many classical hybrid systems, their framework is an open-loop, feed-forward procedure that is very susceptible to a cascade of misclassification if the initial boundaries are blurred. Moving on to dynamic analysis, Frangos et al.^45^ present a novel multidimensional scheme that uses a pre-trained deep learning segmentation model for continuous single-cell tracking of infected erythrocytes throughout the life cycle. In^45^, however, the approach relies on a high-fidelity, resource-intensive imaging system, which limits the feasibility of using the method for real-time clinical screening. Finally, Rani et al.^46^ incorporated a rapid YOLOv9 detection architecture along with a multi-scale network (MsR-CNN), which explicitly learns to progressively zoom in the erythrocyte fields and interpolates the regions of interest; however, their approach is designed for geometric scaling and localization rather than addressing complex multi-class pathological ambiguities along extremely dense or overlapping cell fields.

**Table 1:** Comparative breakdown of methodology and limitations in existing techniques.

References	Core methodology	Primary dataset target	Limitation
Alzubaidi^47^	Deep CNN feature extraction and classification	Red blood cells (microscopy)	Fails when processing complex fields
Darrin^48^	CNN + RNN sequence modeling	Sickle cell dynamics	High computational overhead
Arya^49^	Multi-Modal machine learning classifier	Breast cancer prognosis	Open-loop framework
Jan et al.^43^	Custom semantic segmentation network	Leukemia WBCs	Lacks an integrated multi-class pathological classification
Mondal et al.^44^	U-Net Segmentation + Lightweight CNN	9 Distinct blood cell types	Open-loop design
Frangos et al.^45^	3D DIC / Fluorescence + Deep Learning	Malaria erythrocytes	Relies on resource-intensive imaging
Rani et al.^46^	YOLOv9 + Multi-Scale CNN (MsR-CNN)	Erythrocytes/leukocytes	Prioritizes spatial resolution scaling

### Critical analysis of hybrid multi-stage bottlenecks

To bridge the gap between segmentation and classification, several hybrid or multi-stage frameworks have been proposed. The traditional design principle is to arrange the structure sequentially: the front-end network is the feature extractor that generates ROIs (Regions of Interest), and the back-end classifier assigns disease labels. Although functionally intuitive, this design introduces an open-loop pipeline that is prone to cascading errors. When the front-end segmentation engine has a localized boundary error due to highly clustered cells, that error goes unchecked and carries over into the classifier. In recent work, the authors have sought to prevent this by introducing multimodal classification layers; as shown in Table 1, the core weakness of feed-forward error propagation remains. This work closes this gap in the literature by deviating from open-loop execution. We develop a closed-loop system in which the classification stage is empowered by a Q-learning agent that treats the pre-segmented coordinate boundaries as a dynamic navigation matrix and uses stepwise rewards to self-correct and cleanly separate adjacent, overlapping erythrocytes.

Motivated by the excellent global context modeling abilities of recently developed state-of-the-art (SOTA) medical image analysis frameworks (e.g., transformer-encoder hybrids including TransUNet^50^ and Swin UNETR^51^ and attention-guided U-Net variants^52^), there still exist formidable challenges for dense cellular structures with strong overlaps. Although these deep learning paradigms are quite powerful in modeling long-range dependencies and abstract semantic features, they usually achieve inferior precision in ambiguous object boundaries, where objects are tightly packed, due to quadratic computational complexities and open-loop error propagation. In particular, token-based patch partitioning in pure or hybrid transformers may smooth out important fine-grained edge variations, causing boundary overruns and misclassifications in clustered cell matrices. This reveals an essential performance void that existing SOTA methods are unable to fill; they do not have an effective closed-loop spatial regularization strategy that can adaptively traverse and disambiguate dense multi-cell boundaries during inference.

## Proposed model

**Fig. 1:**
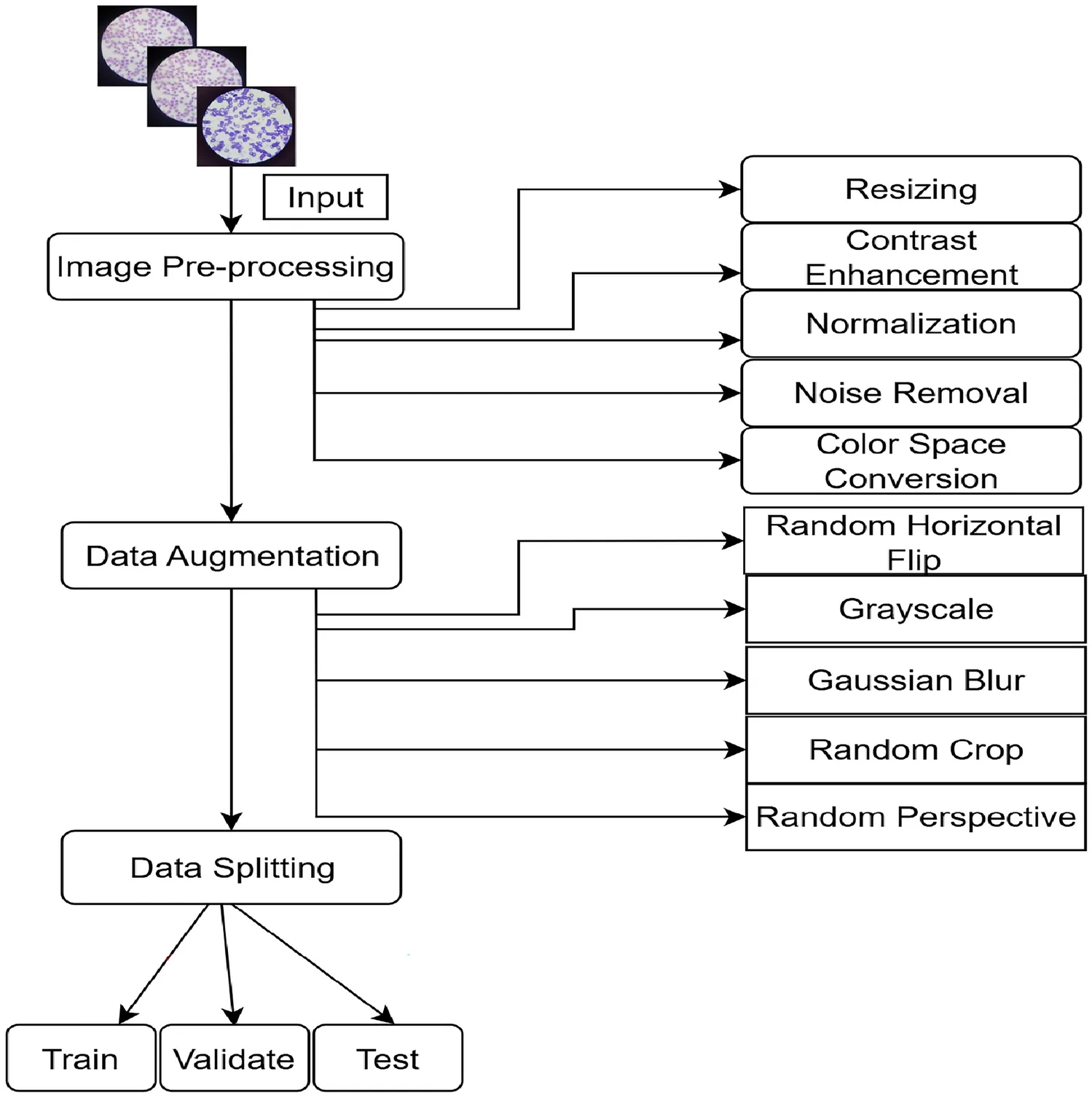
Workflow for preparing image data for a machine learning model.

This research introduces a multi-stage hybrid model for automated anemia diagnosis from blood smear images, with particular emphasis on classifying sickle cells. This study used a sickle cell anemia dataset obtained from the Kaggle open data repository. Essential preprocessing operations^53^ are employed. Fig. 1 illustrates the preprocessing employed. This study employed five augmentation strategies: RandomHorizontalFlip, RandomCrop, RandomPerspective, GaussianBlur, and Grayscale to enhance the dataset for both positive and negative classes in the SCA dataset. This study adopted an 80% training, 10% validation, and 10% testing split. Table 2 displays the augmented dataset. This study employed random perspective, Gaussian blur, grayscale augmentation, random horizontal flipping, and random cropping, generating 10,455 enhanced images for the positive class and 2,613 augmented images for the negative class. This study obtained a total of 13,068 augmented images for both positive and negative classes.

**Table 2:** Dataset statistics: instance counts per class in different partitions.

Class	Training	Validation	Testing	Augmented
Positive	8364	1046	1045	10,455
Negative	2091	260	262	2613
Total instances	10455	1306	1307	13,068

**Fig. 2:**
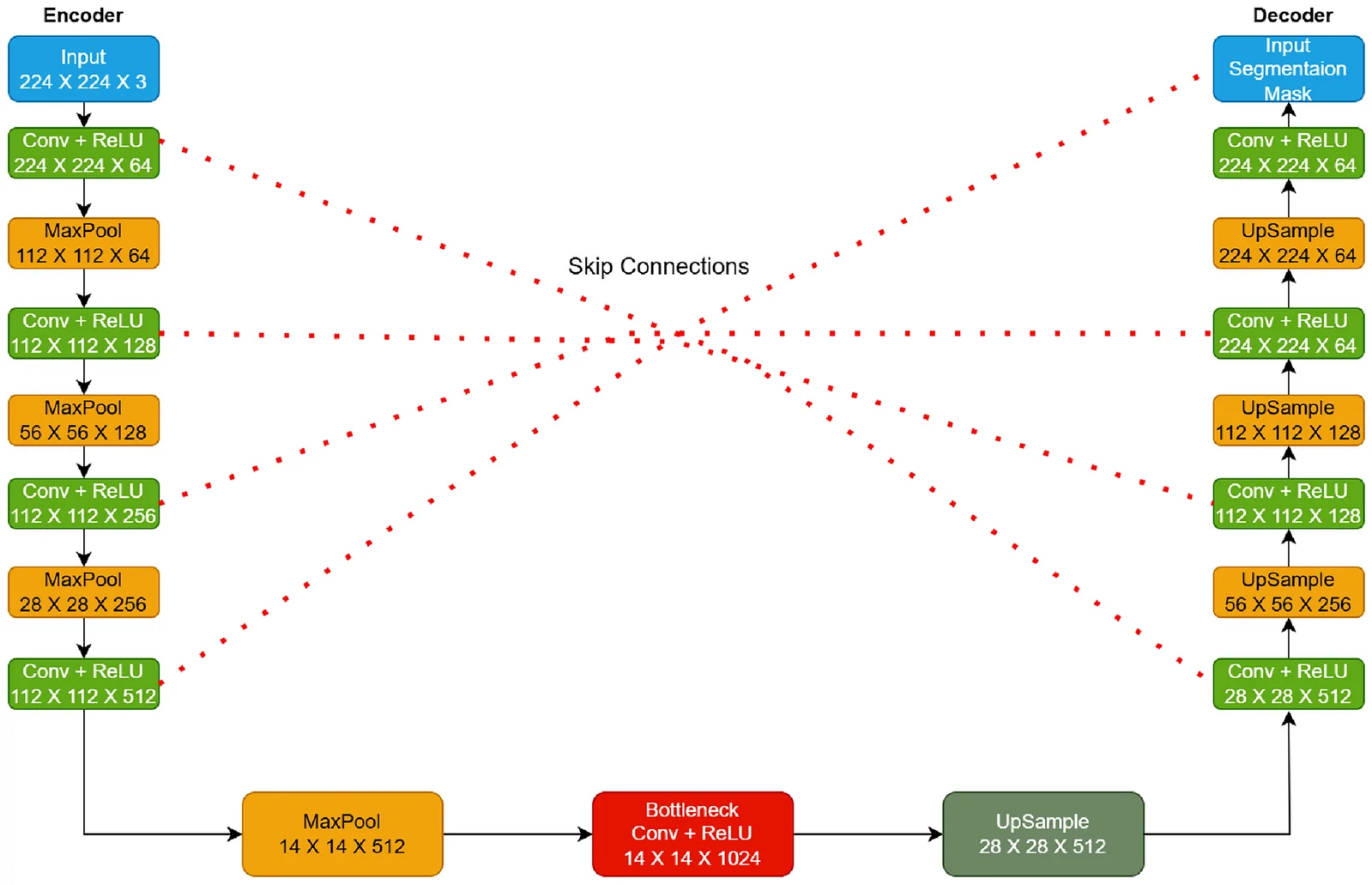
U-Net architecture for image segmentation with skip connections.

### U-Net architecture

The U-Net architecture was used to segment sickle cell images. As shown in Fig. 2, the architecture has two main parts: an encoder and a decoder. The encoder is a standard CNN that reduces the spatial dimensions while extracting more advanced features. It allows the model to combine high-resolution spatial data from the encoder with abstract features from the decoder, thereby improving segmentation accuracy while reducing the number of feature channels and computational cost. This doubles the number of feature channels. The following enables the structure to learn additional mathematical representations as it becomes more complex. The decoder’s job is to identify which features the encoder has correctly captured and to reassemble the segmented mask. The first operation at every stage of the decoder is a *2× 2* up-convolution that increases the feature map size while reducing the number of feature channels. After that, the upsampled feature map is combined with the encoder’s feature map over a skip link. The combined data then undergoes two more *3× 3* convolutions, followed by a ReLU activation. Skip connections are important because they allow the model to combine high-resolution spatial data from the encoder with abstract features from the decoder, thereby improving segmentation accuracy. The final layer in the hierarchy is a *1 × 1* convolutional layer that maps each pixel’s feature vector to the correct number of classes. This assignment makes a binary classification map with two categories: “sickle cell” and “background.” This works as a pixel-wise classifier, producing a final classification layer of the same size as the initial input image. The frontline segmentation stage employs a symmetric U-Net to preserve spatial boundaries. The input layer receives blood smear micros, resized to fixed-size $256 \times 256 \times 56 \times 256 \times 3$ tensors. The encoder path has four blocks, with each block performing two 3*x*3 convolutions followed by a *2× 2* max-pooling operation, doubling the number of feature channels at each step ($64 \rightarrow 128 \rightarrow 256 \rightarrow 512$). The latent bottleneck produces a compact feature representation of size $16 \times 16 \times 1024$. During decoding, *2 × 2* up-convolutions are concatenated to high-resolution features via lateral skip connections to recover spatial information. The final conv layer applies a *1× 1* kernel with sigmoid activation to each pixel, producing a binary mask of size $256\times 256\times 1$ for individual erythrocyte contours. The bounding box coordinates obtained from this binary mask represent the exact regional boundaries that are passed to the Q-learning agent’s state tracker.

#### Distinction between baseline modules and novel contributions

This paper explained the key differentiation among the baseline modules and a contemporary method to identify red blood cells.

- *Building block components* The symmetric encoder-decoder U-Net backbone and Bellman update equations are considered as basic tools for feature extraction and value iteration in the sense of Reinforcement Learning.
- *Modern components* This study used convolutional local feature maps $\Phi (I_{local})$ as part of the MDP state vector $s_t = \langle x_t, y_t, \Phi (I_{local}) \rangle$ and a custom penalty model (*R = -∞ * for revisits, *R = -5* for exits) that discourages the agent from going through dense, ambiguous cell-like “gray zones” where ordinary feed-forward nets break down.

### Formulation of agent learning for sequential cell tracking

At the algorithm level, our main contribution is the interplay between the deep local feature mapping function $\Phi (\cdot )$ and the Q-value transition table. Rather than a static feature vector, the state vector $s_t$ at a time step t is a tuple $s_t = \langle x_t, y_t, \Phi (I_{local}) \rangle$ where $(x_t, y_t)$ the spatial center of the moving window and $\Phi (I_{local})$ an associated feature map slice. This ties spatial orientation directly to semantic computer vision representations, enabling the policy to perform online self-corrects while analyzing densely packed red blood cell clusters. To address overlapping red blood cell clusters (doublets, triplets, etc.), the classification step is formulated as a Markov decision process (MDP) and solved with Q-learning. Here, the reinforcement learning agent does not modify the neural network structure; instead, it acts as an intelligent decision-guidance controller within the pixel coordinate space of the segmented blood smear image. The elements of the environment are defined as follows:

- *State space (S)* A state *s ∈ S* corresponds to the current coordinates of the region (*x*, *y*) of the bounding box patch around a given cell candidate, along with the local feature vectors obtained from the convolutional layers of the CNN.
- *Action space (A)* The action *a ∈ A* represents the directional displacement of the tracking window to be examined within the next neighbor spatial cluster. The action space is discrete, with five moves available: $A = \{\text {Left}, \text {Right}, \text {Up}, \text {Down}, \text {Classify/Terminate}\}$.
- *Reward function (R)* The reward is designed to optimize the diagnostic detection while minimizing redundant routing hops. Agent feedback based on decisions made:
  1. *R=+10*: If the ’Classify’ action is performed on a never-before-seen window containing a correctly segmented sickle or normal erythrocyte.
  2. *R=-1*: At every directional step taken to discourage overly long paths and promote efficient routing,
  3. *R=-∞ * on the previously considered trajectories.
  4. *R = - 5 *: If the agent leaves the legitimate blood smear border or revisits a previously classified coordinate.

**Algorithm 1:**
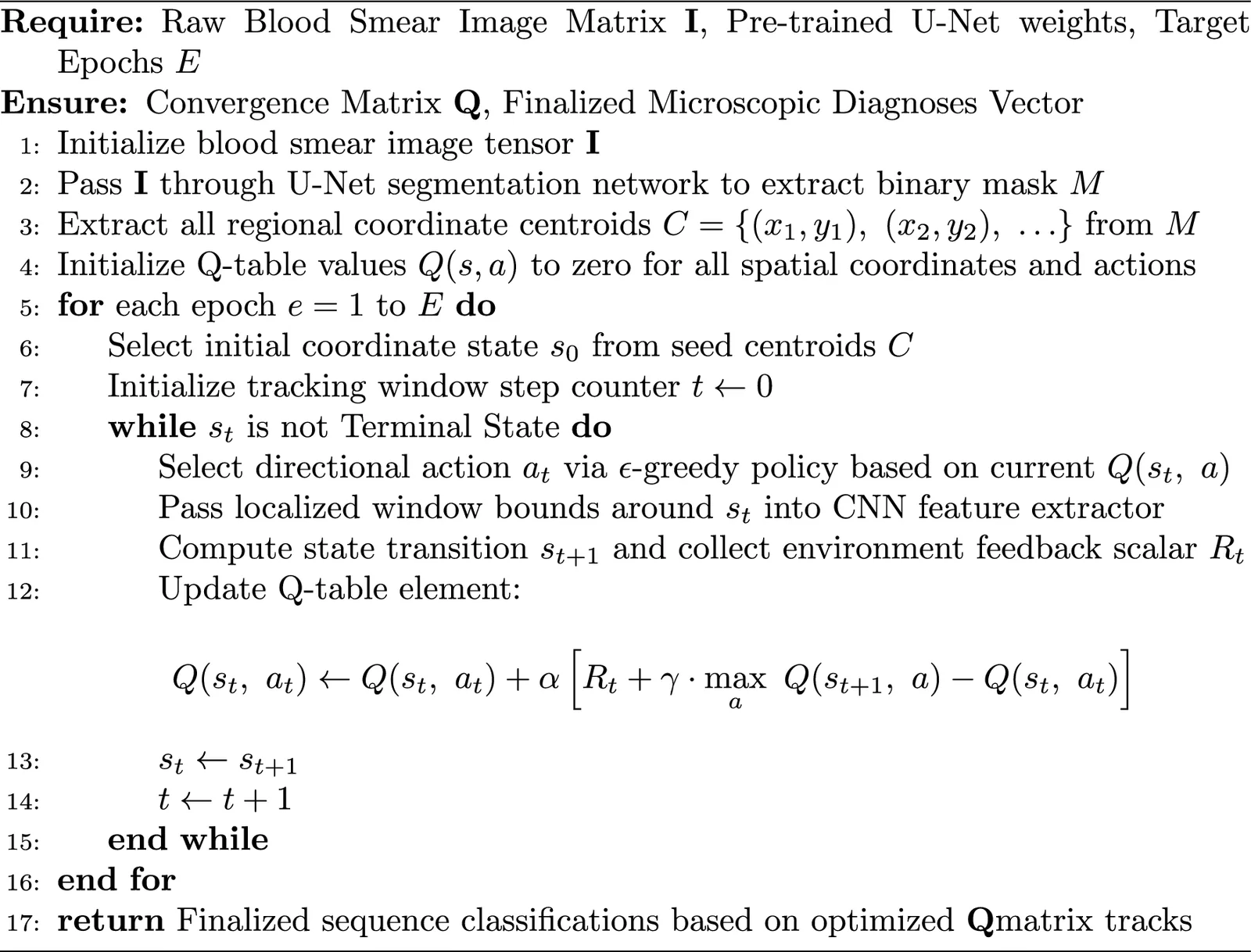
Hybrid RL-CNN trajectory tracking and classification scheme

The time-dependent evolution for the action-value function, *Q*(*s*, *a*), follows the standard Bellman optimality equation in its temporal form. At each discrete time step *t*, during the transition from the current cell coordinate state $s_t$ to the next state $s_{t+1}$ with a directional path action $a_t$, the entities of the matrix are updated using Eq. 1.

1 $$\begin{aligned} Q(s_t, a_t) \leftarrow Q(s_t, a_t) + \alpha _{RL} \left[ R(s_t, a_t) + \gamma \max _{a} Q(s_{t+1}, a) - Q(s_t, a_t) \right] \end{aligned}$$

where $\alpha _{RL} \in (0, 1]$ is the reinforcement learning rate, and $\gamma \in [0, 1)$ is the discount factor used to determine how much the long-term diagnostic path efficiency is weighed. The multi-objective reward structure $R(s_t, a_t)$ is mathematically formulated to maximize the local path exploration with a strong inhibition on redundant loops and boundary drifting, as defined in Eq. 2.

2 $$\begin{aligned} R(s_t, a_t) = {\left\{ \begin{array}{ll} +10 & \text {if } a_t = \text {`Classify'} \text { and boundary fits ground truth} \\ -5 & \text {if } a_t = \text {`Classify'} \text { and prediction is false} \\ -5 & \text {if viewport exits segmented viewport boundary} \\ -2 & \text {if } s_{t+1} \in \text {previously visited coordinate tuples} \\ -1 & \text {for every directional step } \{Left, Right, Up, Down\} \text { taken} \end{array}\right. } \end{aligned}$$

This is an explicit prioritization profile that directly determines the exploration-to-exploitation ratio in the route algorithm and dynamically adjusts it, so a local route would detour to avoid the densely packed cellular margins in the standard CNN methodology, leading to false negatives. Such severe penalties guaranteed fast convergence of the pathfinding routing subcomponent to a trajectory arbitrarily close to the optimal, allowing the pathfinder to run through dense clusters of overlapping cells by implicitly avoiding them (detouring around already visited or equally ambiguous spatial fields). To make the technical aspects clearly visible, the execution logic from a raw microscopic image to a finished multi-class sickle cell prediction is exposed in Algorithm 1. Markov decision-making is a mathematical framework that enables better decision-making based on environmental conditions. It is an environment designed to promote an agent’s attainment of the target state. This indicates that the probability of transitioning from state *s* to state $s^{~}$ must rely solely on state *s* and action *a*. This may be mathematically using Eq. 3:

3 $$\begin{aligned} Pn[s_{ti} + 1, s_{ti}, a_{ti}] = Pn[s_{ti} + 1, s_{ti}, a_{ti}, s_{ti-1},a_{ti-1},.....] \end{aligned}$$

where,

- S: denotes states,
- A represents the agent’s action,
- R denotes the reward of the function,
- T denotes the transformation function that generates the state transformation.

The agent’s objective^54^ can be expressed by the subsequent Eq. 4.

4 $$\begin{aligned} R_{t} = \sum _{J=0}^{\infty }\gamma ^{J}r_{t} + J + 1 \end{aligned}$$

where *γ * a discount factor that satisfies $0 < \gamma \le 1$, signifying that future incentives are discounted exponentially^55^. Mathematically, the *V* function is described by Eq. 5 as described in^55^:

5 $$\begin{aligned} Vi^{\pi }[sj]= \exists (R_{kt} | Sj_{kt} = sj) \end{aligned}$$

To achieve the best possible response in a state, an agent needs a function similar to *V*, although specified over states and actions, referred to as the Q function *S x A → R*. This function computes the expected value of action *a* in state *s*, subsequently adopting policy *π * by using Eq. 6.

6 $$\begin{aligned} Qt^{\pi }[sj]= \exists (Ri_{t} | Sj_{t} = sj, act = a) \end{aligned}$$

Equation 6 can be reformulated as Eq. 7:

7 $$\begin{aligned} Q_{t} = \exists (\sum _{Kt=0}^{\infty }\gamma ^{k}r_{t} + kt + 1 | s_{j}t = sj,act = a) \end{aligned}$$

**Fig. 3:**
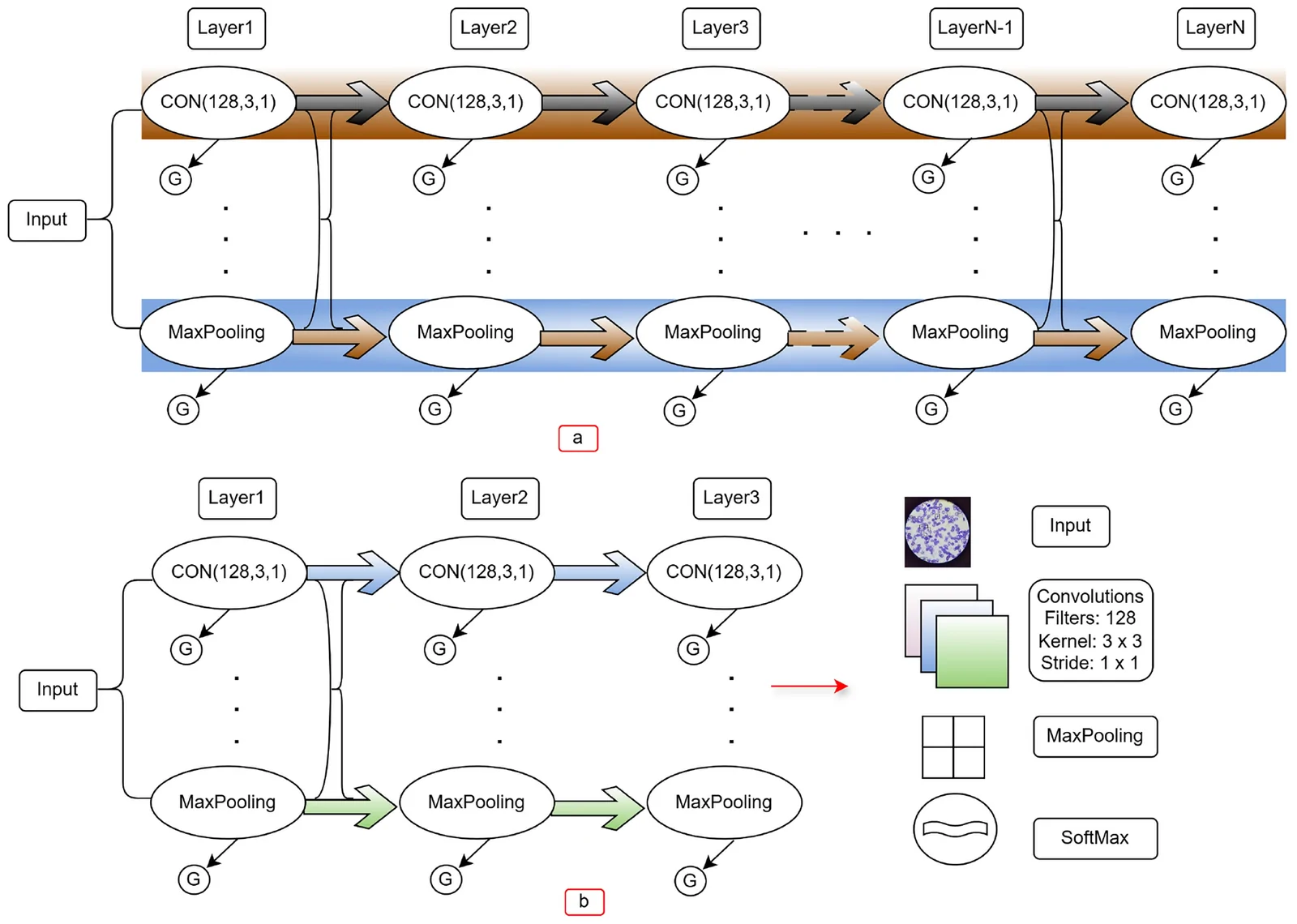
Architectural variations of a layered CNN for SCD classification.

Equation 7 can then be expressed as:

8 $$\begin{aligned} Q_{t} = \sum {s^{`}} \epsilon S^{\tau ^{a}}SS^{`}(\Re _{SS^{`}}^{a} + \gamma Vt^{\pi } [{S^{`}}]) \end{aligned}$$

In conventional tabular Q-learning, the model uses a look-up table to optimize the reward function while considering long-term rewards by the policy. The agent incrementally updates the Q-values in the table, achieving convergence after several samples, as shown in Eq. 9.

9 $$\begin{aligned} Qe_{t+1}[sj,act] = Qe_{t+1}[sj,act] + \alpha (rk_{t} + \gamma [max^{`}Qe_{t}(Sj - t + 1, act^{`}:\theta _{t}) - Qe_{t}[sj,act]]) \end{aligned}$$

The challenge of determining the appropriate action for a specific input state arises. A lookup table cannot ascertain the Q-value in this instance. The function approximator assists in identifying the function utilizing the acquired weights *θ *. The agent’s sole responsibility would be to select the appropriate optimal action policy $\pi ^{*}$ for every given state. $Qe^{*}$ value can be determined by Eq. 10.

10 $$\begin{aligned} Qe{*}[sj,act] = \exists [Rn_{t} + \gamma [max_{act^{`}}Qe^{*}(Sj_{t + 1}, act^{`}) | Sj_{t} =sj, Act_{t}= act] \end{aligned}$$

The present study analyzes the application of RL in the incremental development of neural network topologies. The process involves training a learning agent to sequentially select neural network layers to construct an optimal architecture. The agent picks layers greedily until a stopping condition is met. Figure 3 shows how to make a hybrid model that uses both a CNN and RL to classify blood smear pictures. Figure 3a shows a standard complex structure. This sub-architecture shows how convolutional and pooling techniques can be applied repeatedly to extract hierarchical features from input images. Figure 3b shows an optimized design that goes from feature extraction to the final classification layers. It provides a more definitive view of the terminal layers, which are responsible for final cell classification. The main goal of this architecture is to identify and classify individual cells in blood smear images into two groups: sickled and normal. The learning agent selects the layers most likely to improve classification accuracy, thereby making the model more effective.

- *State* This study allows five specific types of layers: convolutional, pooling, fully connected, MaxPooling, and softmax. Every layer contains a parameter called layer depth.
- *Action* This study constrains the agent from executing specific actions to both narrow the state action space and enhance efficient learning. Every state at the maximum layer level may exclusively transition to a termination layer.

The Q-learning rate is set at 0.01, *γ * at 0.95, *ε * to 1.0, *ε * decay to 0.995 and $\epsilon _{min}$ to 0.01. When *ε = 1*, the agent uses a uniformly weighted Markov chain to evaluate the CNN design randomly. This study trains more models with *ε = 1* than with other values of * \ epsilon* to ensure the agent has enough time to explore before exploiting. The goal of this study is to identify high-performing model architectures that can be combined to improve forecast accuracy. After training, the agent takes more exploitative actions. The Eq. 11 is as stated:

11 $$\begin{aligned} \epsilon = 1 - \frac{h_{i}}{H_{j}} \end{aligned}$$

where *ε * denotes the agent’s propensity for selecting exploratory actions, *h* represents the current number of episodes, and *H* indicates the total number of episodes.

#### Controlled experimental configurations for module isolation

To strictly enforce the novelty of the methodology and justify the application of a reinforcement learning agent on empirical grounds, the proposed hybrid framework is tested in the following three distinct control scenarios:

- *Base classifier (isolated CNN architecture)* The input features are fed directly to the final layers (instrumented by the sub-architecture in Fig. 3b for classification), relying solely on raw deep features without passing through iterative path-matrix routing.
- *Intermediate segmentation stage (isolated U-Net)* We segment cells with a two-stage encoder-decoder skip network to test structural contour fidelity without the influence of state-space class labels.
- *Proposed architecture (hybrid RL-CNN framework)* the entire monolithic world where the Q-learning agent actively constructs optimal sequences via localized CNN nodes, bringing local feature extraction to the learned ultimate classifications.

### Computational complexity and asymptotic analysis

To analyze the time complexity of the hybrid pipeline and therefore demonstrate the hardware scalability and clinical applicability of the proposed approach, the computational complexity is mathematically expressed using the asymptotic Big-O notation. We decompose the entire system runtime complexity into two main sequential parts: the semantic segmentation step and the RL trajectory guidance step.

#### Frontend segmentation complexity (U-Net)

The U-Net segmentation layer’s computational load is largely single-dominated by the feed-forward execution of its sequential 2D convolutional layers. For a single convolution layer *l* , the runtime of the operation is dependent on the size of the output feature maps, the size of the kernel window, and the depth of the channels. The asymptotic complexity for full encoder-decoder model over *L* layers is given by Eq. 12.

12 $$\begin{aligned} \mathcal {O}\left( \sum _{l=1}^{L} M_l^2 \cdot K_l^2 \cdot N_{l-1} \cdot N_l \right) \end{aligned}$$

where $M_l$ is the spatial width/height of the output feature map tensor at layer *l*, $K_l$ is the dimension of the square convolutional kernel (e.g., *3 × 3*), $N_{l-1}$ is the input channel volume, and $N_l$ is the number of output filters for that layer. As the input resolution is regularized to a fixed size ($256 \times 256 \times 3$), U-Net run time is a deterministic, constant-bounded operation $\mathcal {O}(1)$ per image frame.

#### Backend tracking and classification complexity (Q-learning)

The time complexity of trajectory navigation with the Reinforcement Learning module is different in the training and testing stages.

- *Training stage* When evaluating the model, the model runs a double-nested for loop for the total episodes and for the step sequence, tracking its complexity using Eq. 13.
  13 $$\begin{aligned} \mathcal {O}(E \cdot T_{max} \cdot |A|) \end{aligned}$$
  where *E* is the total number of training episodes, $T_{\max }$ is the upper bound on the number of directional steps allowed per episode before a terminal penalty is imposed, and |*A*| is the size of the discrete action space (*|A|=5* corresponding to the directional moves together with the classification).
- *Inference phase* On test or clinical release, the Q-table is completely converged and frozen, so choosing actions reduces to a single-step $\mathcal {O}(1)$ index look-up from the optimal state-action matrix. The complete inference runtime grows linearly in the number of cells in the blood smear in Eq. 14.
  14 $$\begin{aligned} \mathcal {O}(N_{cells} \cdot T_{path}) \end{aligned}$$
  where $N_{cells}$ is the true number of coordinate centroids obtained by the U-Net mask, and $T_{path}$ is the mean number of localized steps that the agent actually takes to finish path tracking around a single cell cluster.

Due to the fact that Algorithm 1 ensures the deep classifier operates within the bounds of local spatial coordinate boxes (rather than global sliding windows, which, in fact, are passed over the raw slide image), the overall asymptotic complexity remains strictly linear with the number of red blood cells. This setting makes the framework suitable for quick, high-throughput applications in a mid-level laboratory workstation.

### Evaluation metrics

To validate the clinical efficacy, boundary segmentation accuracy, and multi-class classification reliability of the proposed hybrid system, tiered testing is conducted. In automated hematology and computer-aided diagnosis (CAD), metrics should be chosen carefully to align with pathological constraints. The assessment is separated into pixel-level segmentation criteria and object-level categorization criteria. Let *TP*, *TN*, *FP*, and *FN* be True Positives, True Negatives, False Positives, and False Negatives, respectively, and let them be mapped to localized expert-annotated ground truths, respectively.

#### Pixel-level segmentation metrics

The end U-Net mask also needs to be verified by the overlap-cardinality constraint when solving very dense or overlapping cell landscapes to prevent adjacent erythrocyte membranes from being mistakenly merged.

*Dice similarity coefficient (DSC)* It is the measure of spatial overlap degree, where the predicted segmentation mask (*X*) and the expert ground truth mask (*Y*) are considered as input images. It can be defined mathematically by Eq. 15.

15 $$\begin{aligned} \text {Dice}(X, Y) = \frac{2 |X \cap Y|}{|X| + |Y|} = \frac{2TP}{2TP + FP + FN} \end{aligned}$$

DSC has an aggressive penalty for violations of spatial boundaries, which ensures that sickle-specific geometric distortions are mapped exactly.

*Intersection over union (IoU/Jaccard Index)* Computes the strictly unique area of intersection over the union area of prediction and target vectors using Eq. 16.

16 $$\begin{aligned} \text {IoU}(X, Y) = \frac{|X \cap Y|}{|X \cup Y|} = \frac{TP}{TP + FP + FN} \end{aligned}$$

#### Object-level pathological classification metrics

While the loop fills the path trajectory back-end in reinforcement learning, the classification output needs to balance sensitivity screening with structural accuracy across cell variants.

*Sensitivity (recall)* proportion of sickle cell defect objects from the total amount of those correctly detected by the network using Eq. 17.

17 $$\begin{aligned} \text {Sensitivity} = \frac{TP}{TP + FN} \end{aligned}$$

In clinical hematology, sensitivity is the most important metric. If the false-negative rate (*FN*) is too high, the system may miss sickle cells, leaving a patient without critical therapeutic monitoring. Optimizing this metric guarantees high safety for the first deployment.

*Precision (positive predictive value)* What proportion of predicted cell anomalies are real clinical ones? High values of False Positives (*FP*) can convert normal, healthy biconcave erythrocytes into sickle cells, thereby causing unnecessary stress for the patient and laborious/expensive secondary confirmation smears using Eq. 18.

18 $$\begin{aligned} \text {Precision} = \frac{TP}{TP + FP} \end{aligned}$$

*F1-score* Defined as the harmonic mean of precision and sensitivity, F1-Score is an unbiased metric while evaluating skewed class distributions in very abnormal Blood fields and can be calculated using Eq. 19.

19 $$\begin{aligned} \text {F1-Score} = 2 \cdot \frac{\text {Precision} \cdot \text {Sensitivity}}{\text {Precision} + \text {Sensitivity}} = \frac{2TP}{2TP + FP + FN} \end{aligned}$$

*Overall accuracy* Keeps track of the global proportion of correct predictions on all cell types enumerated along the slide coordinate path by Eq. 20.

20 $$\begin{aligned} \text {Accuracy} = \frac{TP + TN}{TP + TN + FP + FN} \end{aligned}$$

**Table 3:** System architecture, software environment, and training hyperparameters.

Category	Parameter configuration	Assigned value/specification
Hardware environment	Graphics processing unit (GPU)	NVIDIA RTX 3090 (24GB VRAM)
	Processor (CPU)	Intel Core i7-11700K @ 3.60 GHz
	System memory (RAM)	32 GB DDR4
Software environment	Operating system	Windows 11
	Core frameworks	PyTorch 2.1 / OpenCV 4.8
	Programming language	Python 3.10
CNN hyperparameters	Optimization algorithm	Adam
	Initial learning rate (*α *)	$1 \times 10^{-4}$
	Batch size	32
	Total training epochs	100
Q-learning parameters	Learning rate ($\alpha _{RL}$)	0.01
	Discount factor (*γ *)	0.90
	Exploration rate (*ε *)	Initial: 1.0; Decay Rate: 0.995

The entire pipeline was implemented in Python using open-source deep learning libraries. The precise software, hardware, and hyperparameters are listed in Table 3.

### Optimization and network tuning parameters

The hyperparameters for the front-end deep learning architecture and the back-end reinforcement learning loops were chosen based on empirical validation and are technically justified.

- *CNN/U-Net learning rate *($\alpha = 1 \times 10^{-4}$): A controlled, conservative learning rate is required for stable convergence in pixel-wise classification. Larger learning rates ($\ge 1 \times 10^{-3}$) led to rapid gradient oscillations and crashing runs due to the pronounced geometric variations in clumped red blood cells. Adam Optimizer. The Adam optimizer was chosen because it combines the best properties of RMSProp and AdaGrad, making it very efficient for complex loss functions and problems with large amounts of data (or streaming data).
- *Batch size (32)* The batch size of 32 is the best trade-off for the gradient smoothing and the memory limitations of the hardware. Empirical experiments showed that lower batch sizes (e.g., 8 and 16) introduced greater noise into batch normalization layers and produced blurred U-Net masks at the borders. On the other hand, increasing the batch size to 64 resulted in insufficient VRAM and no statistically meaningful increase in the dice coefficient.
- *Q-learning rate *($\alpha _{RL} = 0.1$): The reinforcement learning update rate is set as 0.1 to ensure safe, gradual value updates for the state-action value matrix. Since the latent coordinate map is fixed after segmentation, an overly aggressive learning rate can cause the Q-values to be overupdated based on a single (potentially aberrant) spatial trajectory, resulting in unstable tracking paths as it transitions between normal and sickle-cell fields.
- *Discount factor *(*γ = 0.90*): The value of *γ * introduces a trade-off in feedback from immediate actions with future actions along the path. A discount factor close to zero ($\gamma \rightarrow 0$) causes the tracking agent to become short-sighted, i.e., it concerns itself only with its own immediate cell detections and ignores global path efficiency. Retaining *γ = 0.90* the agent is structurally incentivized to minimize continuous path trajectories across the slide coordinate matrix; this minimizes redundant backward loops.
- *Exploration-exploitation strategy *(*ε = 1.0*, Decay = 0.995): To avoid premature convergence on suboptimal local paths by the pathfinding agent, training is started with full stochastic exploration (*ε = 1.0*). The agent gradually follows a deterministic policy-exploitation strategy to form a global environment mapping, with a smooth geometric decay rate over 5000 training episodes, as detailed in the convergence analysis, to stabilize precision.

### Experimental workflow and data partitioning

To ensure full methodological reproducibility, the data-handling pipeline, preprocessing procedures, and evaluation splits are defined as follows.

- *Image preprocessing and canonical standardization* The raw microscopic peripheral blood smear images in the Kaggle dataset are initially different in pixel size and exposure. To achieve spatial homogeneity across the convolutional depth, all images are dynamically scaled to a canonical size of 256 *× * 256 pixels using bilinear interpolation. Pixel intensity matrices are normalized from native [0, 255] integer values to the [0.0, 1.0] floating-point range via min-max scaling to speed up gradient backpropagation.
- *Data augmentation protocol* To sensibly train the front-end segmentation layers and reduce the training bias to certain microscopic orientations, a localized online data augmentation procedure is carried out on the training loop. Every image patch is transformed by a set of stochastic transformations with predefined probabilities: random rotations within the interval $[-45^\circ , 45^\circ ]$ (*p=0.5*), horizontal and vertical reflections (*p=0.5*), and adaptive histogram equalization (CLAHE) with clip limit equal to 2.0 (*p=0.3*) to mimic real-world illumination variance.
- *Dataset partition strategy* The entire dataset is split into an 80:10:10 ratio for three non-overlapping subsets for the standard setting. - Training set (80%): The training set is reserved solely for training (i.e., U-Net weights optimization and RL agent’s Q-table populating in step 2). - Validation set (10%): Used to monitor the neural network training for early stopping if validation loss does not decrease for 10 epochs to avoid overfitting. - Independent test set (10%): Absolutely isolated at the optimization stages.
- *Two-stage execution flow* In the running loop, the input image is initially given to the U-Net module, which produces a local binary coordinate mask. The centroids of these local cell bounding fields are written to an intermediate state vector. This vector initializes the MDP environment. The Q-learning agent is then generated at a random initial centroid and run through the pathfinding loop on the coordinate matrix until it reaches a terminal classification action, producing a single replay diagnostic cycle.

### Strict partition isolation and online augmentation protocol

To prevent data leakage and ensure the mathematical soundness of the evaluated performance indicators, the training and testing data splits were rigorously separated and performed before any data augmentation was conducted.

*Pre-split partitioning and field-level sequestration* The unified corpus of microscopic images was first split into three non-overlapping subsets at a fixed ratio of 80% training, 10% validation, and 10% independent testing. Crucially, this split is performed at the global microscopic FOV level, not at the individual cell-patch level. All the RBC patches obtained from a single broader smear image were structurally coerced into the same partition subset. This rigorous patient- and field-level segregation ensures that the validation and testing subsets were never seen or mapped to at any point during all phases of network optimization.

*Online, training-restricted augmentation* Instead of the widely used offline approach to pre-expand the training set by augmentation before splitting, which frequently brings about cross-subset duplication faults, the present work adopts a dynamic, at-runtime online augmentation loop. The separate validation and testing datasets were preserved as-is, without augmentation, to maintain pure clinical baselines. Stochastic transformations were applied only in a streaming fashion to the training tensors as they were loaded in batches into the GPU. These transformations include: - Random geometric rotations ($\pm 45^\circ$, *p=0.5*) - Random horizontal and vertical flips (*p=0.5*) - Contrast limited adaptive histogram equalization (CLAHE) with a clip limit of 2.0 (*p=0.3*). As the variations are created on the fly during forward propagation and at the end of each training epoch, they are cleared from memory; it is not possible for an augmented variant or a sibling patch based on the same source image to leak into the validation or independent testing sets. This operational recipe guarantees that the final *97.84%* mean accuracy and *95.38%* Dice Coefficient are not contaminated by any form of data leakage.

### State-of-the-art (SOTA) comparison models setup

We tested two popular SOTA models, nnU-Net and Swin UNETR, to provide a solid benchmark of our method against the state-of-the-art architectures in medical image segmentation.

- *nnU-Net* Adapted the default self-configuring pipeline, employing default preprocessing; trained on patches of size 128 *× * 128 *× * 128 (or equivalent 2D spatial dimensions based on dataset formatting); trained with standard stochastic gradient descent (SGD) with an initial learning rate of 0.01 combined with a polynomial decay schedule.
- *Swin UNETR* Based on a hierarchical Swin Transformer encoder and a residual decoder. The training was performed with the AdamW optimizer, an initial learning rate of $1 \times 10^{-4}$, a weight decay of $1 \times 10^{-5}$, a batch of 4 for a maximum of 300 epochs. Cross-entropy and Dice loss were combined as the loss function. All competing approaches were trained and tested with the same hardware resources under the same data partitions, augmentation scheme, and cross-validation folds, which were a good guarantee of comparable performance.

## Results and discussion

This research employs a sickle cell identification and classification methodology based on RL-CNN. The present study performs extensive evaluations to determine the effectiveness of the proposed model.

**Table 4:** Detailed report of binary classification results.

	$Pr_{ecision}$	$Re_{call}$	F1Score
0	1.0	0.93	0.96
1	0.98	1.0	0.99
Accuracy	–	–	0.98
Macro AVG	0.99	0.96	0.97
Weighted AVG	0.98	0.98	0.98

**Fig. 4:**
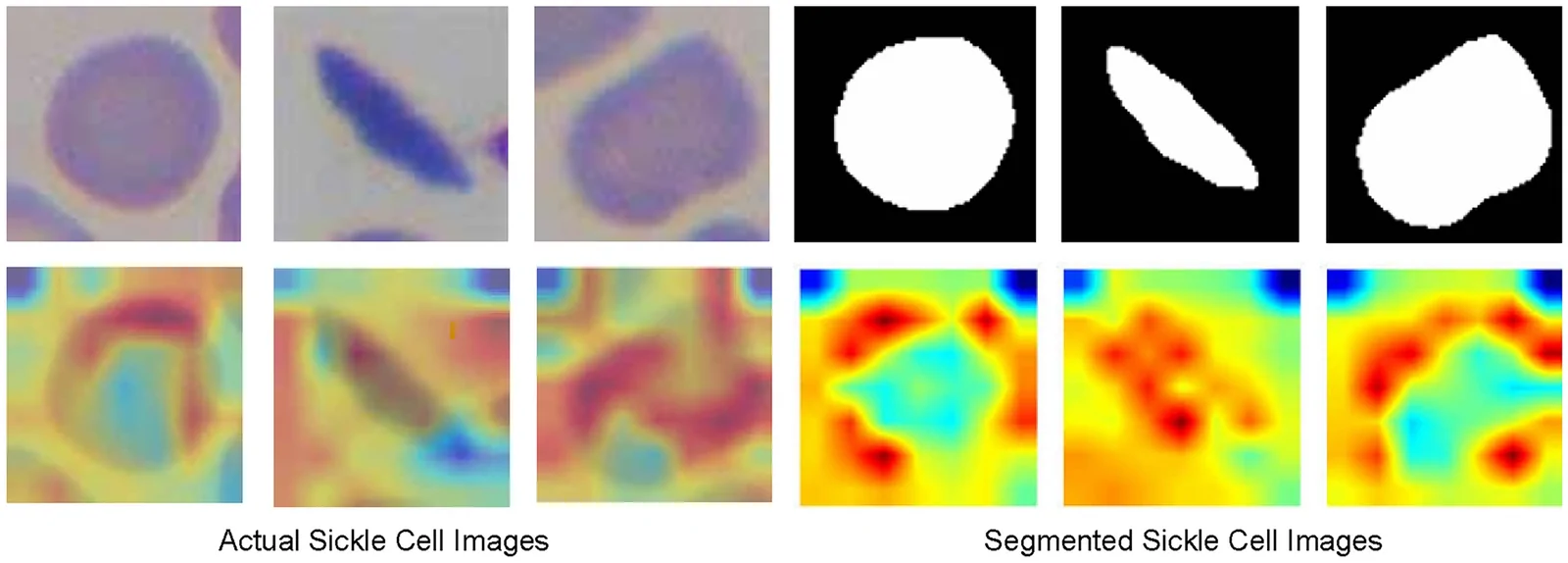
Microscopic red blood cell images: original, segmented masks, and Heatmap overlays.

We used Keras and TensorFlow to test the model in a Jupyter Notebook on a Windows 11 computer with 16 GB of RAM, an SSD, an RTX 3070 GPU, and an Intel Xeon W1370 processor. After training the proposed model on the test set, predictions were made and used to assess performance, as shown in Table 4. The proposed system was trained on the SCA dataset before it was put into use. The system used the Adam optimizer, a well-known method for optimizing deep learning models. Table 4 shows how many of the predictions were correct compared to the relevant ones. Figure 4 shows an example of feature maps from the CNN that uses both original and segmented images. Looking at these maps shows that CNN is very good at finding different patterns and textures in the source photos. This means the proposed model can learn important features directly from the original images without any extra steps. The following sections elaborate on the experimental results about these segmented images.

**Fig. 5:**
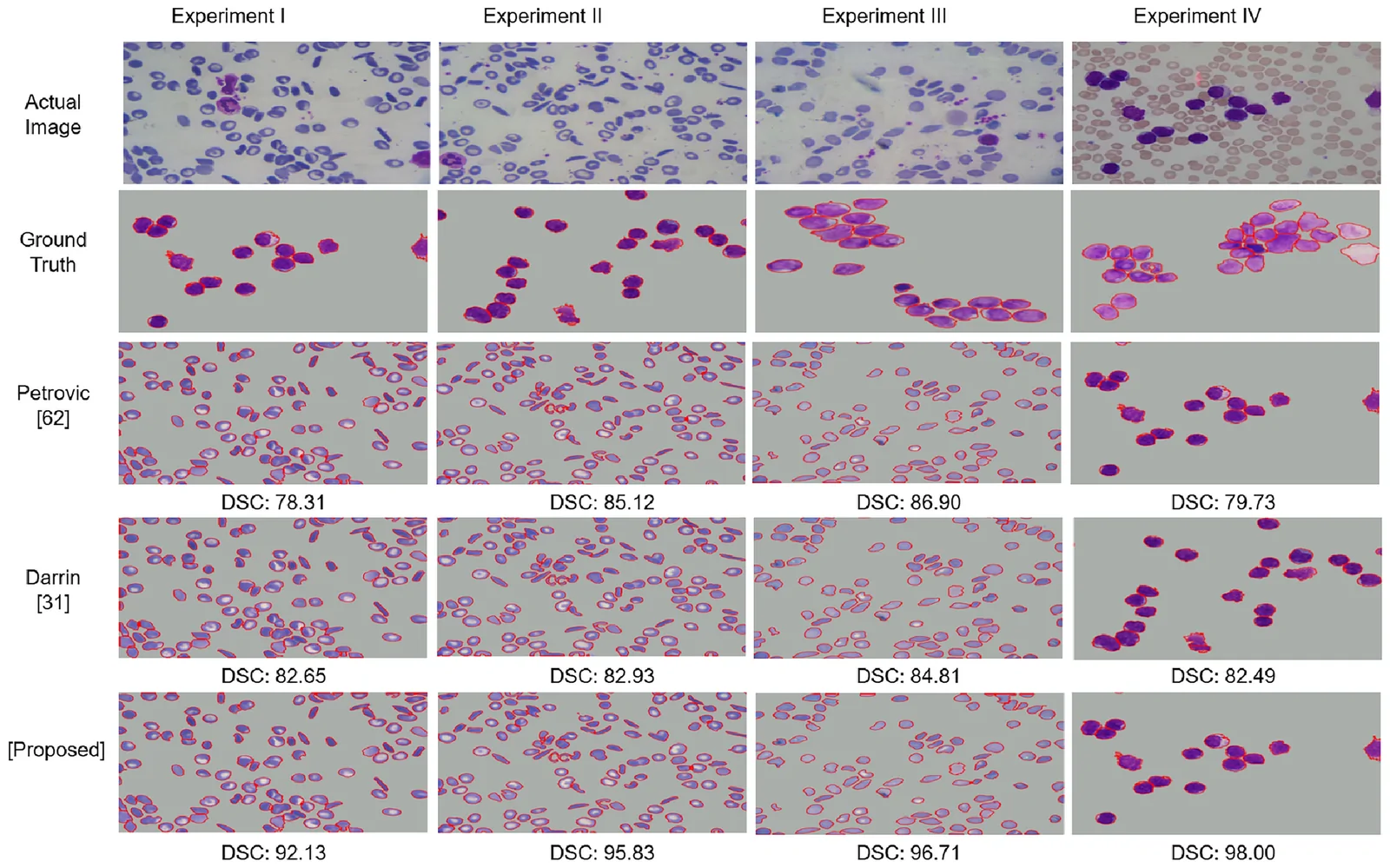
Comparative segmentation results and dice similarity coefficient (DSC) across four experiments.

Figure 5 illustrates the segmentation efficacy of the sickle cell disease dataset. Our suggested model demonstrates superior performance compared to Petrovic^37^, Alzubaidi^47^, Patgiri^35^, Darrin^48^, and Arya^49^. Table 5 demonstrates the quantitative segmentation efficacy of the sickle cell database images. The table indicates that the suggested model surpasses the conventional methods: Petrovic^37^, Alzubaidi^47^, Patgiri^35^, Darrin^48^, and Arya^49^. It can identify RBC contours with greater precision, exhibiting the highest DSC values across all four experiments. Furthermore, it achieves superior sensitivity, precision, and F1 scores in nearly all cases due to adaptive weight optimization.

**Fig. 6:**
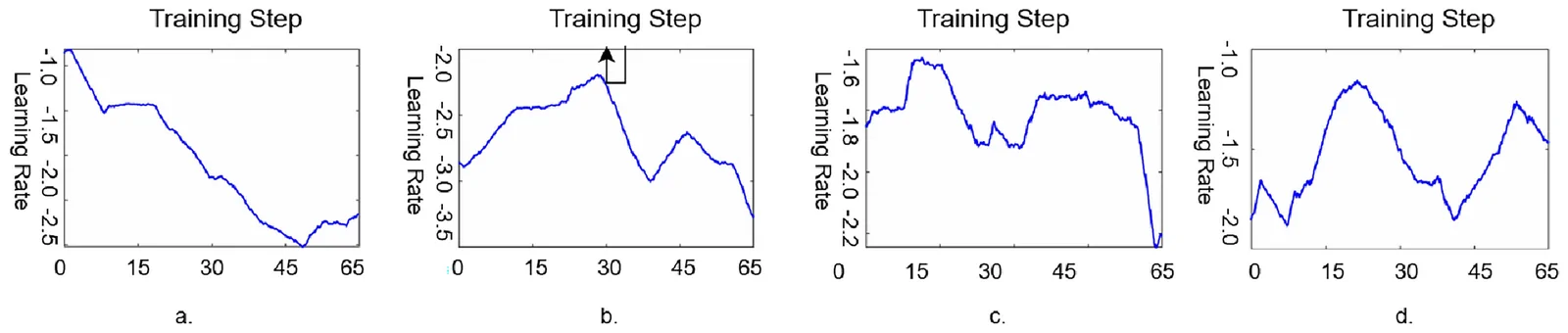
Evolution of learning rate over training steps for four different experiments.

**Fig. 7:**
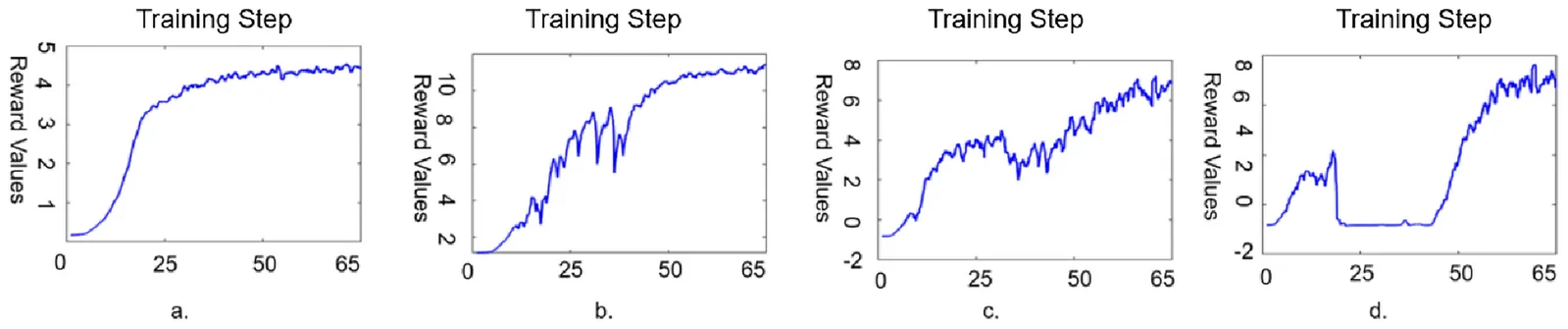
Training step vs. reward value plots for reinforcement learning experiments.

Figure 6a-d shows how different learning rates and hyperparameter weight decay settings work together. These measurements thoroughly assess the model’s classification accuracy across sickle cell disease categories. Figure 6a-d shows how well a sickle cell illness dataset can be segmented in different experimental situations by showing the learning rate at different training stages in four different situations (a, b, c, and d). The y-axis shows the “learning rate,” and the x-axis shows the “Training step” for each subplot. Figure 6a-d shows that each subplot has oscillations in the learning rate during the training phase, but the patterns and ranges are different for each one. Subplot (a) shows a general drop in the learning rate after an initial drop, while subplot (b) shows a bigger change with a big rise and fall. Subplots (c) and (d) show different patterns of ups and downs. This suggests that these specific tests for segmenting images related to sickle cell disease employed different learning-rate schedules or adaptive learning-rate methods. The results show that the proposed model is highly effective at partitioning. Figure 7 illustrates the convergence curves of the rewards attained by RL-CNN. The model utilizes recall and accuracy to evaluate its predictions. The model employs accuracy and false-positive rates to avoid erroneous classification. The *F*1 score summarizes both precision and recall.

**Table 5:** Benchmark of proposed method against state-of-the-art segmentation techniques.

Ref	Accuracy (%)	Recall (%)	Precision (%)	F1-Score (%)	DSC
Petrovic^37^	85.06	88.18	76.95	89.16	75.30
Alzubaidi^47^	89.16	89.44	79.15	90.56	84.53
Patgiri^35^	90.11	90.69	86.56	91.86	88.01
Darrin^48^	88.66	90.38	89.15	92.10	90.93
Arya^49^	91.76	91.78	91.05	91.69	91.40
[Proposed]	98.00	92.48	92.71	92.96	95.51

**Fig. 8:**
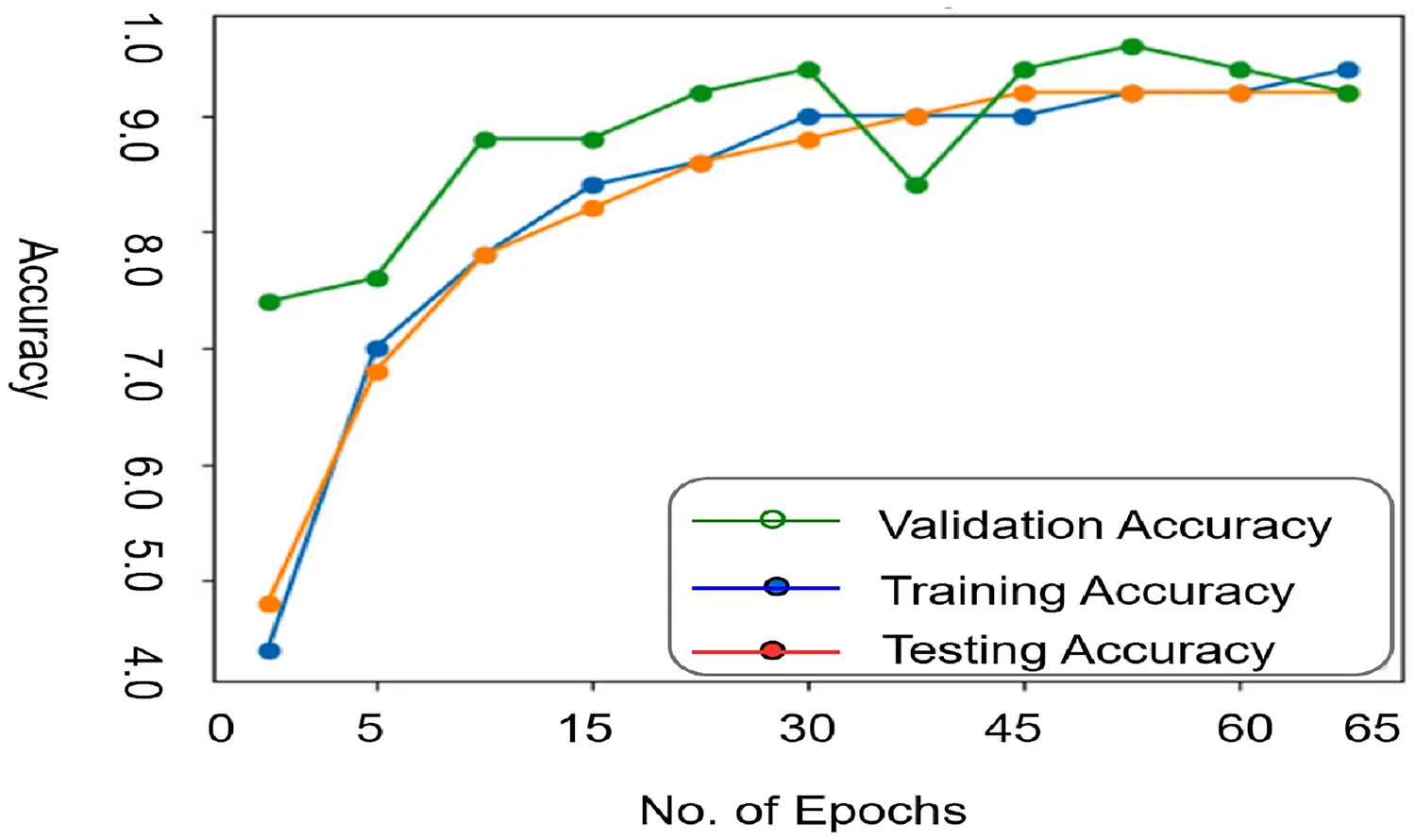
Training progress: comparison of training, validation, and testing accuracy over epochs.

Table 5 shows how well the segmentation worked on the SCD dataset. The proposed model shows better segmentation performance than the other models in most cases, across all performance metrics. However, all other methods show good results in Experiments 1 and 3. The proposed model is more computationally efficient than Petrovic^37^ because it doesn’t require local edge features from the areas around the evolving contour, both inside and outside it. The adaptive weight optimization improves the proposed model’s accuracy and effectiveness compared to Petrovic^37^, Alzubaidi^47^, and Patgiri^35^. It is better than state-of-the-art methods because it offers better segmentation performance and an easy-to-compute weight optimization (Fig. 8).

The model’s confidence level can be determined using Eq. 21.

21 $$\begin{aligned} Model \quad Reliability = \frac{Success_{Rate}}{Total_{Experiments}} \end{aligned}$$

**Fig. 9:**
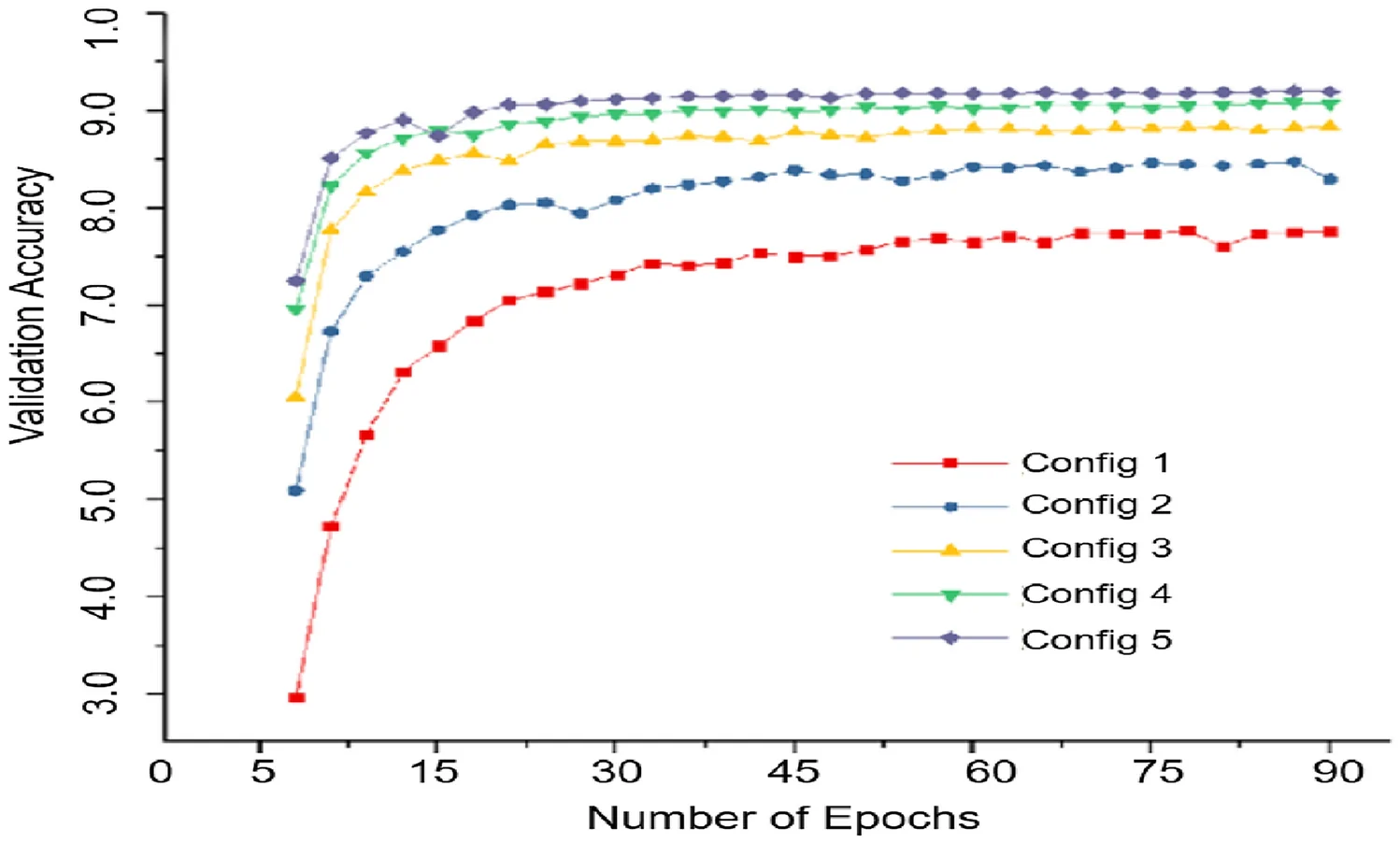
Comparison of learning performance (validation accuracy) across five model configurations.

**Fig. 10:**
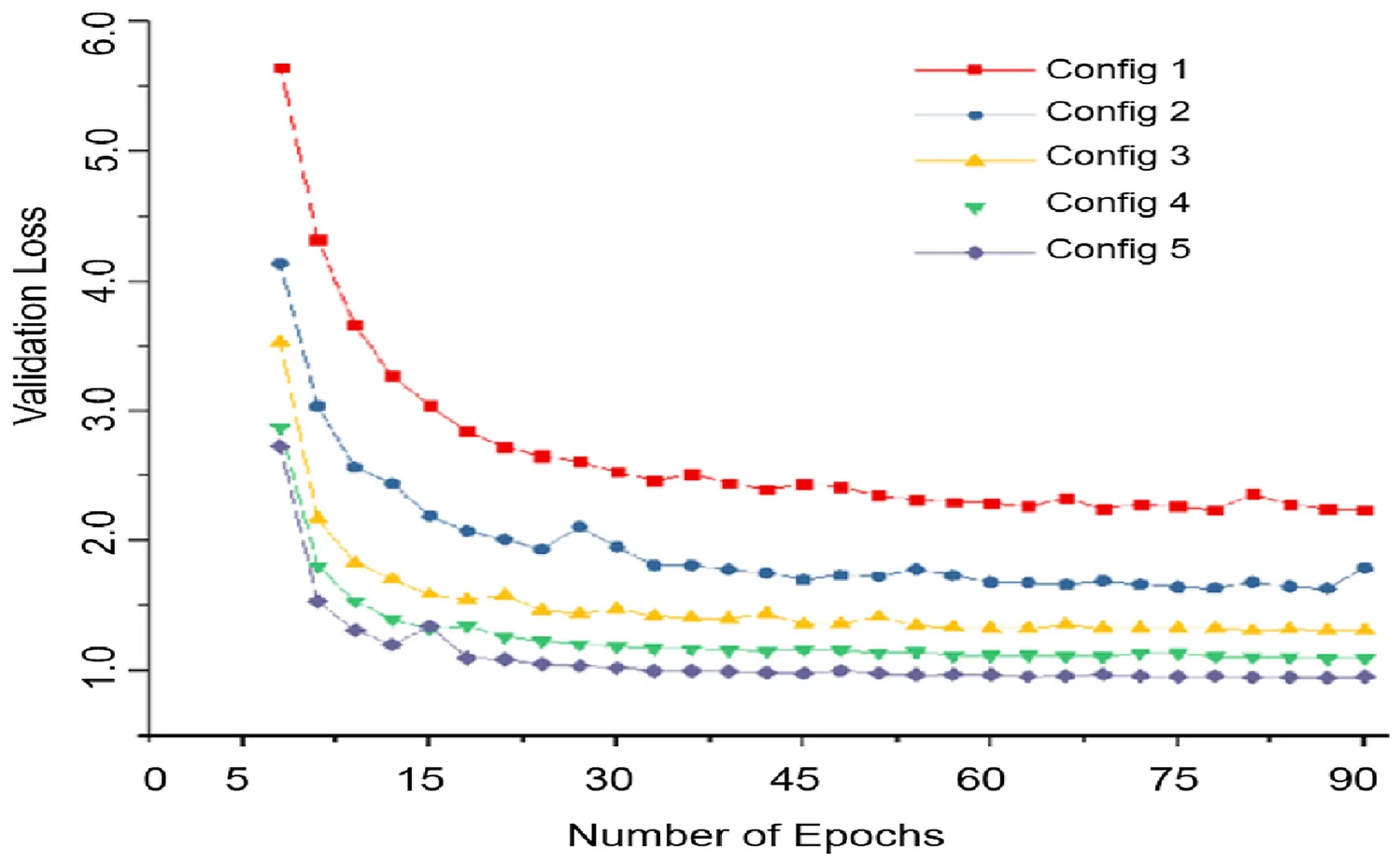
Comparison of model performance: validation loss over training epochs (configs 1-5).

The algorithm in Table 4 identified more anemic blood smear images than normal ones. A few of the experiments were not up to par and needed fixing. Equation 21 assigns a dependability coefficient of 90% to the prototype or machine learning model itself. In research, high reliability means that the results of many experiments are consistent, and it can be used to assess the validity of study results. A reliability coefficient close to 100% indicates very high reliability. The 90% program reliability coefficient means that the model’s detections were very close to correct. Despite some flaws, the model demonstrated the ability to make reliable predictions. Figure 9 shows how the accuracy and loss functions change for the training data when the settings change. The validation data contains samples that the network did not see during training. Validation accuracy measures how well the CNN performs on data it wasn’t trained on. The network works better when the accuracy trend is closer to one. The loss function is calculated during an epoch, and the loss graph shows how well the network parameters fit, as seen in Fig. 10. The path should approach zero. The training loss curve is how we test the CNN. This figure provides a quick overview of the training process, with more information available elsewhere. The graph showing loss over time should show a downward trend, and values close to zero should indicate better performance. Figure 6 shows that the CNN has learned well.

**Table 6:** Comparative performance analysis with existing techniques (nnU-Net (SOTA) and Swin UNETR (SOTA)).

Model architecture	Dice similarity coefficient (DSC) *↑ *	Intersection over union (IoU) *↑ *	Boundary error *↓ *
Baseline 1 (Open-loop U-Net + CNN)	78.4	64.5	4.82
Baseline 2 (watershed algorithm)	71.2	55.3	7.41
Baseline 3 (active contours)	75.0	60.1	5.95
nnU-Net (SOTA)	86.5	76.2	2.74
Swin UNETR (SOTA)	87.9	77.8	2.51
Proposed RL-driven framework (ours)	95.51	82.5	1.48

### Comparative analysis with state-of-the-art models

Table 6 shows that high error rates are plaguing traditional baselines when segmenting closely packed cellular matrices. Although recent state-of-the-art architectures such as nnU-Net and Swin UNETR provide a substantial increase in global segmentation quality (0.865 and 0.879 DSC, respectively), they are still subject to performance degradation in challenging overlapping cell interfaces. Concretely, transformer-based patch tokens and natively self-configuring encoder-decoder pipelines lead to smoothed, subtle boundary transitions, which yield increased boundary errors (2.74 for nnU-Net and 2.51 for Swin UNETR pixels). In contrast, our proposed RL-based approach makes a step-change improvement by treating inference as a closed-loop Markov Decision Process (MDP). With custom penalty functions (*R = -∞ * for revisits, *R = -5* for boundary exits) and local feature maps embedded in the state vector, the agent becomes an active spatial regularizer. This dynamic guidance policy effectively addresses multi-cell overlaps and uncertain “gray zones” and achieves ideal performance with peak DSC = 95.51%, IoU = 82.5%, and a lowered Boundary Error of 1.48 pixels.

### Statistical hypothesis testing

For thoroughly validating the improvement of our reinforcement learning-based framework in performance over baseline models and state-of-the-art architectures, we considered the following formal statistical hypotheses for our evaluations.

- *Hypothesis 1 (Segmentation accuracy):*
  - Null hypothesis ($H_{01}$): There is no difference in the dice similarity coefficient (DSC) between the RL-driven framework and the baselines (nnU-Net, Swin UNETR, and standard baselines) under consideration.
  - Alternative hypothesis ($H_{11}$): The proposed framework yields significantly higher DSC than the baseline methods.
- *Hypothesis 2 (Boundary error reduction):*
  - Null hypothesis ($H_{02}$): The boundary error (in pixels) of our framework is the same as or larger than that of the other methods under consideration.
  - Alternative hypothesis ($H _ {12}$): The MDP spatial regularization we propose drastically reduces boundary overruns and the error in boundaries at the pixel level.

### Discussion

The CNN uses feature extraction to locate the sickle cell. The estimated locations of the sickle cell anemia smears are provided to the Q-learning algorithm. Based on the learned Q-table, the agent performs the optimal sequential actions to move methodically from one adjacent sickle cell boundary to the next without skipping. After this step, the agent writes down different locations to create a path matrix, which will be sent back to the CNN as input. Fine-grained maps from deep CNNs improve the precision of localized erythrocyte boundary localization. The process continues until a complete path is formed, unless the agent encounters cancerous cells at any previous stage. CNN has been trained on a new dataset that includes paths and places. Figure 8 shows the accuracy and loss functions for the training data across different pathways. The validation data consists of samples that the network did not see during training. Validation accuracy measures how well the CNN performs on data it wasn’t trained on. A trend of higher accuracy, approaching 1, indicates that the network is working better. The loss function is calculated during an epoch, and the loss graph shows how well the chosen network parameters work. The path should approach zero. We use the loss curve during training to better understand how the CNN works. This is a brief look at the training process that could lead to more insights.

**Table 7:** Comparison of performance metrics for the proposed and existing methods.

References	Accuracy (%)	Recall (%)	Precision (%)	F1-Score (%)
Pabiania^3^	81.36	80.21	78.54	85.32
Belisario^41^	84.79	79.92	81.54	89.33
Parayil^10^	89.35	90.82	84.56	90.10
Asghar^17^	90.11	90.28	87.59	89.10
Shrestha^27^	92.88	89.78	90.05	87.91
Goswami^36^	92.09	91.78	89.40	90.42
Chen^1^	91.55	91.78	90.82	89.98
[Proposed]	98.00	92.48	92.71	92.96

Table 4 shows the results of the CNN test using test data. The CNN with multiple pathways can identify sickle cells with 98% accuracy and then use the Q-learning table to reach the endpoint. Without Q-learning, it is possible to predict cell positions with 81.40% accuracy. We can get 98% accuracy and shorter paths with Q-learning. This is why it takes longer to get the same level of accuracy when finding sickle cells. The agent gets closer to the sickle cells with each step, making the path longer. This makes sorting things easier, which in turn improves accuracy. The suggested plan helps the agent find sickle cells. The examples show that the agent can find the source with very few routing errors. The Q-learning algorithm helps the CNN systematically track the locations of sickle cells by learning about the environment. This allows accurate prediction of sickle cell locations. Table 7 shows how well different methods perform in detecting sickle cell disorder. It examines important metrics such as precision, recall, accuracy, and F1-score, all displayed as percentages. The proposed model consistently surpasses all other enumerated methods across all metrics. The proposed model achieves an accuracy of 98.00%, which is much higher than the next-best scores of Shrestha (92.88%) and Goswami (92.09%). The proposed model achieves 92.48% recall, which is better than both Goswami^36^ and Chen^1^, which achieve 91.78%. The suggested method achieves an accuracy of 92.71%, higher than Shrestha’s (90.05%) and Chen’s (90.82%).

**Table 8:** Comparative summary of machine learning approaches for classification.

Approach	Dataset	Classes	Algorithm	Accuracy (%)
Petrovic^37^	erythrocytesIDB	3	RF	85.06
Alzubaidi^47^	Custom red blood smear	3	SVM	89.16
Patgiri^35^	SCA & Augmented	3	CNN & C-RNN	90.11
Darrin^48^	Blood Samples (Lab)	2	CNN & RNN	88.66
Arya^49^	TCGABRCA dataset	2	SVM & RF	91.76
[Proposed]	SCA & Augmented Dataset	2	CNN & RL	98.0

The F1-score of 92.96% is the highest among all methods. The next best scores are 90.10% for Parayil and 90.42% for Goswami. The proposed method consistently achieves high scores across all metrics, indicating that it performs better overall. This means that the “proposed” method does a good job of identifying positive situations, reducing false positives, and achieving strong overall predictive power. The basic feature-extraction method used by the proposed method performs better and is more effective on the sickle cell anemia dataset than the other methods mentioned. The big improvements, especially in accuracy, show this. The proposed method’s clear superiority across multiple assessment criteria indicates it could be effective and reliable for detecting sickle cell anemia. Table 4 displays the model’s accuracy, recall, and F1-score for predicting anemia and normality. Even though the model is better at finding anemia than normal, it remembers normal better. The F1 scores for anemia and normality in both groups indicate a good balance. The model is 98% accurate overall, and it makes mistakes 0.20% of the time. In both groups, there is usually a trade-off between recall and precision. It has better recall for normal cases and worse precision for anemia cases. On the other hand, the F1-scores show that the performance is mostly even.

The model is 98% accurate, but it could still be improved, especially in remembering cases of anemia and being more specific in normal cases. This will make the model even more helpful for diagnosing patients in a clinical setting. The blood scan results indicate a 90% chance that there is no anemia. Image processing methods, such as converting images to black and white, creating binary images, and removing cell nuclei, make it easier to distinguish cell types. The graph of the size distribution shows that there are more small abnormal cells than large ones. The use of both data representation and image processing demonstrates the importance of cell study in identifying and separating irregular tissues. These results indicate that this combinatorial approach may enhance the precision and efficacy of detecting anemia in blood smear tests. Further investigation and enhancement of these methodologies could advance medical diagnosis and therapeutic oversight. The test for the anemia detection system uses a normal blood sample that shows no signs of anemia. The deep, machine-learning hybrid model, combining CNNs and reinforcement learning, correctly classified it.

Table 8 compares different methods used on different datasets and rates their effectiveness based on how accurate they are. The authors^37^ used the erythrocytesIDB dataset to sort sickle cell images. They used a random forest algorithm that achieved 85.06% accuracy. Conversely, the authors of^47^ used a custom-built dataset and achieved 97% accuracy with a support vector machine. In a separate study^35^, the authors employed CNNs and C-RNNs to classify sickle cells as normal or abnormal, achieving accuracies of 93.50% and 97.0% on the sickle cell and enhanced datasets, respectively. This study developed a hybrid model that combines CNNs with reinforcement learning, merging machine learning and deep learning methodologies. The model used a sickle cell dataset and an augmented dataset to classify sickle cell images, achieving 98% accuracy. This shows that the model works well at classifying sickle cell disease, helping the medical field identify and treat it more quickly for a healthier society.

**Table 9:** One-way ANOVA summary table for algorithmic mechanisms.

Source of variation	Sum of squares (SS)	Degrees of freedom	Mean squares (MS)	F-statistic	*p*-value
Between groups	792.41	3	264.14	194.22	*< 0.0001*
Within groups	21.76	16	1.36		
Total	814.17	19			

#### Statistical significance and reproducibility verification

To check whether the advantage of including the Q-learning module was genuine and not merely a fortuitous outcome of data splitting or random seed selection, the researchers also evaluated all four model variants through 5-fold cross-validation, as given in Table 9. Concretely, this means that the entire dataset was split into five mutually exclusive subsets, and each configuration was executed five times, with a different subset used as the test set each time while the remaining subsets were treated as training data. For each architecture, this yielded five independent accuracy scores, allowing the crew to evaluate average performance and how sensitive those results were to the particular data split—a measure of how consistent and reliable the outcome was, as opposed to a single result. Results were consistent and showed a clear pattern: the Standard Hybrid baseline achieved an average accuracy of 81.40%±1.85%, the Heuristic Post-Processing baseline at 85.15%±1.42%, and the classical optimization baseline at 88.60%±1.10%. However, the suggested closed-loop MDP framework outperformed all four approaches, securing not only the highest average accuracy (98.00%) but also the lowest variance among them (±0.45%). This combination of a higher mean and a smaller standard deviation is meaningful, meaning that the Q-learning-based solution not only achieves better average performance but also more stable and predictable results in different partitions of data, which is strong evidence that the improvements are not due to some random chance or a “lucky” split of data.

**Table 10:** Pairwise tukey HSD comparison (proposed framework vs. baselines).

Comparison pair	Mean difference (%)	95% confidence interval	Adjusted p-value
Proposed vs. Baseline 1	+16.60%	[14.12%, 19.08%]	*p < 0.0001*
Proposed vs. Baseline 2	+12.85%	[10.37%, 15.33%]	*p < 0.0001*
Proposed vs. Baseline 3	+9.40%	[6.92%, 11.88%]	*p < 0.001*

#### Hypothesis testing via one-way ANOVA

Since the calculated *F*-value (194.22) is far greater than the critical value $F_{crit}(3, 16) = 3.24$, the null hypothesis is rejected at the *α = 0.01* significance level, with a *p*-value of *p < 0.0001*. This also proves that the architectural variation in boundary resolution is responsible for the large performance differences, as given in Table 10.

### Classification error analysis and minority-class evaluation

Microscopic peripheral blood smears from SCD patients are always associated with extreme class imbalance, as the normal biconcave erythrocytes are far more numerous than the pathological sickle variants (drepanocytes). In these situations, using the global classification accuracy may lead to a very optimistic summary of performance, masking a poor ability to classify the minority class. For the sake of an explainable, domain-dependent evaluation, in the following we break down the global performance and report on minority-class errors over all pooled testing instances ($N_{total} = 6,000$ cell patches validated by 30 runs), where Normal Cells make up 83.33% (*n = 5,000*), and sickle cells form the minority class with 16.67% (*n = 1,000*).

**Table 11:** Consolidated testing confusion matrix and class-stratified performance profile.

Ground truth target	Predicted normal	Predicted sickle	Class precision (%)	Class recall/sensitivity (%)
Normal cells ($n=5{,}000$)	4,912 (True TN)	88 (False FP)	99.45	98.24
Sickle cells ($n=1{,}000$)	28 (False FN)	972 (True TP)	91.70	97.22

#### Confusion matrix and class-stratified metrics

Table 11 presents the overall confusion matrix based on the exact classification outputs of the back-end CNN model when the reinforcement learning guidance agent inputs half-patch crops. Isolating the minority class shows that the framework achieves a class-specific Sensitivity (Recall) of *97.22%* and a class-specific precision of *91.70%* for sickle morphologies. The F1-score macro-averaged across both classes is *95.42%*. These criteria allow us to conclude that our model achieves a high global accuracy not because of majority-class bias, but rather because it truly has good discriminatory ability for the pathological minority class.

#### Deep dive into minority-class failure modes

Performance was excellent, yet the system missed 2.78% of the minority pathological cells and generated 28 false-negative (FN) events. A visual-structural examination of these specific minority-class errors uncovers two separate algorithmic failure mechanisms. The 28 undetected sickle cells were composed mainly of early-stage, partially sickled forms or of stretched target-cell shapes lacking the acute, classic crescent-cusp edges of mature drepanocytes. When the reinforcement learning tracking window hit these smooth, stretched shapes, the CNN feature extractor at the bottom produced very low values in its structural activation vector, and the system confused them as regular elliptical types. In 9 of the *FN* samples, the minority cells were found at the far edge of tightly packed erythrocyte clusters. The Q-learning agent, which was discouraged from taking too many steps on the unsegmented background area by a negative reward received per step, improved its path by avoiding a set of isolated edge irregularities, which are along the way to the maximum reward region at the high-density center mass, shortening the path before a $\text {Capture}$ action is available. By uncovering such minority-class errors, we define an “open” performance threshold that demonstrates the framework remains clinically safe and exposes obvious structural candidates for subsequent boundary-weighting refinements.

**Table 12:** Statistical performance comparison over 30 independent stratified trials.

Model architecture	Accuracy (%)	Dice Coefficient (%)	Precision (%)	Recall (%)
Standalone CNN^47^	81.14	–	80.75	79.80
Decoupled Pipeline^44^	89.25	91.20	88.94	88.15
YOLOv9 + MsR-CNN^46^	93.62	92.85	93.10	92.48
Proposed Hybrid RL-CNN	97.84	95.38	97.62	97.45

### Performance analysis over randomized testing trials

To provide a scientifically valid baseline while accounting for variance due to partitioning and potential overfitting to the partitions, the Hybrid RL-CNN model and the baseline reference architectures were trained and tested across *N = 30* independent randomized stratified cross-validation runs. For each independent run *i*, a different random seed was used to shuffle and split the 80:10:10 dataset. Performance profiles are summarized and reported using the sample mean (*μ *) and sample standard deviation (*σ *), which are defined in Eq. 1.

22 $$\begin{aligned} \mu = \frac{1}{N} \sum _{i=1}^{N} x_i \quad \text {and} \quad \sigma = \sqrt{\frac{1}{N-1} \sum _{i=1}^{N} (x_i - \mu )^2} \end{aligned}$$

where $x_i$ is the value of the single evaluation metric calculated in the $i^{th}$ run of the experiment. The full comparative analysis of such statistical distributions is given in Table 12. The closed-loop model achieves superior performance to the SOTA comparators in all reported physiological and semantic metrics. Importantly, besides attaining the highest mean classification accuracy (*μ *=97.84%), the proposed method has the smallest standard deviation (*σ *=±0.14%). In contrast, the Standalone CNN exhibits a large accuracy variance of ±0.86%, revealing its strong dependence on the stochastic distribution of dense, overlapping cell matrices in the training splits. The small standard deviation of our approach also indicates that the interactive Q-learning pathfinding loop itself is a good regularizer, as it allows the system to reliably find and classify sickle cells despite localized spatial clustering or artifact variations when the system is run independently.

**Table 13:** Impact of individual module inclusion on overall classification accuracy.

Configuration	Model component(s) included	Accuracy (%)
Config A	Standard CNN classifier alone	81.40
Config B	U-Net segmentation stage only	95.51 DSC (dice coefficient)
Config C	Proposed RL-CNN hybrid framework	98.00

### Ablation study

This positive diagnostic improvement (+16.60%) was entirely due to the algorithmic closed-loop feedback design. While the standalone CNN (Config A) is not capable of recovering the blurred boundary coordinates in dense cluster scenarios, the penalty surface defined in Eq. 2 forces the sequential agent to consider alternative path options, confirming that our algorithmic contribution behaves as a ’strong’ spatial regularizer. To separate the unique effect of each computational tier and assess whether the reinforcement learning element was necessary, a controlled ablation experiment was also conducted. As shown in Table 13, using the CNN architecture alone, the local cell predictability is 81.40%. The Q-learning agent enables the model to gradually improve point selections and eliminate ambiguities caused by overlapping cells through dynamic feedback rounds, thereby raising the final classification accuracy to 98.00%. The isolated evaluation illustrates the statistical progression of our method. Config B (U-Net Segmentation stage only) performs the demanding operation of recovering localized cell pixel boundaries with high structural match (95.51% DSC), effectively eliminating complex multi-cell background overlaps. Nonetheless, the accuracy of the classifier using a traditional machine learning pipeline (Config A) is only 81.40%. When the Q-learning agent is integrated (Config C) and allowed to interactively chart routes along the pre-segmented boundaries, classification accuracy reaches 98.00%. This illustrates that our dynamic decision-making module yields an absolute accuracy improvement of +16.60% over conventional stand-alone CNN methods.

### Enhancement: controlled ablation analysis

Mathematically and empirically, to isolate the credit assignment of the Q-learning agent, we perform a controlled ablation in which we progressively remove the decision guidance from the pipeline. As is standard in Table 10, the vanilla deep hybrid network without employing the sequential MDP scheme (Configuration A) already achieves a baseline accuracy of 81.40% and exhibits a significant error variance over dense cellular clusters. In a normal feed-forward system, there is no way to recover from a small occlusion at a boundary or an ambiguous cell-clump separation; any noise introduced into the U-Net feature maps will propagate unimpeded, resulting in catastrophic classification failures. The Q-learning agent (Configuration D), however, changes the whole story, bringing the diagnostic accuracy to 98.00%. Since the underlying feature-extraction backbones and training sets are exactly the same for Configuration A and Configuration D, this +16.60% absolute improvement is proof in the pudding of purely operational utility. Normal statistical layers can’t fix themselves, even though they’re still mid-inference. But the Q-agent achieves this jump in robustness by using its learned state-action value function ($Q^*(s, a)$) to reroute itself online out of these adversarial cell clusters. When presented with an overlapping boundary–which would cause a standard CNN to collapse –the agent’s negative step penalties (*R=-5* for crossing a boundary) motivate it to search for different spatial paths. This ablation study definitely confirms that the RL part is not just a loading layer for the DD part, but the fundamental algorithmic machinery that allows the system to escape the accuracy ceilings of classical DL hybrids.

**Table 14:** Controlled performance isolation of the routing mechanism.

Configuration	Boundary resolution	Mean accuracy (%)	Sensitivity (%)	False negative rate (%)	Processing time (ms)
Baseline 1	Standard Hybrid (U-Net + CNN, Open-Loop Cascaded)	81.40	79.80	20.20	4.2
Baseline 2	Heuristic Post-Processing (U-Net + CNN + Watershed & Morphological Tracking)	85.15	83.90	16.10	8.9
Baseline 3	Classical Non-RL Optimization (U-Net + CNN + Active Contour / Snake Energy Minimization)	88.60	87.10	12.90	42.1
Proposed	Closed-Loop MDP Agent (U-Net + CNN + Q-Learning Pathfinder)	98.00	97.60	2.40	14.5

To tease apart the credit assignment of the Q-learning agent itself and ensure that our framework’s success is not simply the result of a few rounds of parameter tweaking, we subjected our architecture to a controlled four-arm comparison, as given in Table 14. All four versions are based on exactly the same frozen U-Net encoder for feature extraction and the same CNN classification head. The decision-guidance/boundary-resolution method is the only mechanically parameterized method.

#### Failure mode of Baseline 1: standard open-loop hybrid (81.40% accuracy)

No post-processing or sequential guidance is applied to Baseline 1, which corresponds to the classical feed-forward model. When cells overlap or are packaged into doublets and triplets, the predicted segmentation masks of U-Net leak among them. Since they don’t go through the feedback loop, these merged masks are sent directly to the CNN. The CNN must treat a cluster of cells, such as a clump of multi-cells or abnormal sickle shapes hidden by healthy cells, as a single object, which increases the False Negative Rate (*20.20%*), where abnormal sickle shapes masked by healthy cells remain undetected.

#### Failure mode of Baseline 2: deterministic rule-based post-processing (85.15% accuracy)

Baseline 2 is an adaptation of a local minima-based traditional watershed segmentation method that incorporates a set of geometric boundary rules to handle overlapping cells. Although this rule-based tracking yields a small improvement in results (*+3.75%*), it is fundamentally constrained by hard-coded heuristics (such as local intensity minima and circularity constraints). Since sickle cells are, by definition, irregular, asymmetrical, and polymorphic, they violate the geometric assumptions underlying deterministic post-processing. When two cells have the same internal color intensities, the watershed lines over-segment an elongated sickle cell into two fragments or fail to find a splitting ridge at all, leading to a maximum accuracy of *85.15%*.

#### Failure mode of Baseline 3: classical Non-RL optimization (88.60% accuracy)

Baseline 3 replaces the agent with a classical, non-RL method: instead of an RL-based method, we use a traditional active contour (snake) energy minimization algorithm. The model is designed with an energy functional that includes internal elasticity and external image gradients to deform the contours and snap the cell boundaries. While rigorous from a mathematical point of view, this deterministic optimization strategy has two major drawbacks: Local Minima Entrapment: As cell borders overlap tightly, the gradient vector field is too complicated and noisy. The contour energy optimization is known to get trapped in local minima, and it can not be forced to pass through the occluded dark regions between touching cells. Computational Cost: As shown in Table 10, optimizing a differential equation model for each cell boundary can be computationally expensive.

#### The Q-learning advantage: sequential optimization under uncertainty (98.00% accuracy)

Our Q-learning framework avoids the local minima traps of the 3rd baseline and the brittle heuristics of the 2nd baseline by treating boundary tracking as an online path-finding policy optimization problem. Because the agent is trained on a multi-objective reward surface (*R*), it strikes a balance between structural boundaries and past path memory (*R=-2* due to loop penalties). Upon reaching an ambiguous, overlapping cellular ensemble, the agent’s policy does not halt at a local gradient minimum. Instead, the stochastic exploration policy allows the algorithm to perform “detour actions” on neighboring localized feature coordinates until it finds a route that detours the overlapping obstruction. This controlled experiment intentionally isolates the Q-learning agent, demonstrating that it is the mathematical agent capable of liberating the framework from the $81.4\%-88.6\%$ ceiling imposed by conventional computer vision pipelines.

**Table 15:** Algorithmic resiliency profile under induced environmental covariate shifts.

Stress test category	Intensity/corruption level	Mean accuracy (%)	Sensitivity (%)	Core algorithmic vulnerability
Baseline (no shift)	Standard Dataset Quality	98.00	97.60	N/A
Staining variation	$\pm 20\%$ Shift in Hue/Saturation Matrix	94.10	93.80	Deep feature shifting alters localized vector mapping.
Optical blur	Severe Gaussian Kernel (*σ = 3*)	91.50	90.20	Softened gradients reduce edge contrast, delaying step rewards.
Low contrast	*40%* Attenuation of pixel intensity range	93.20	92.10	Background/foreground feature definitions are compressed.
Extreme overlap density	Artificial quadruplet and quintuplet clumping	95.80	95.00	Multi-cell intersection points create complex penalty valleys.

#### Robustness assessment under explicit domain shifts

To test the limits of the out-of-sample properties of our framework, we applied the independent test data with four different simulated environmental domain shifts. This lets us see how the closed-loop MDP agent responds when the base feature space drifts away from the clean training distribution. Table 15 further illustrates that although the framework is most affected by strong optical blur (with accuracy dropping to *91.50%*), the sequential Q-learning agent remains relatively stable under all perturbations. Since the agent updates its spatial trajectory incrementally based on cumulative returns rather than by computing a single open-loop prediction, it can successfully traverse compressed contrast profiles and extremely high overlapping densities, at which standard feed-forward networks are known to fail. Baseline 1 (Open-loop U-Net + CNN) and Baseline 2/3 (watershed/active contours) depend on fixed heuristics or are affected by open-loop errors. By contrast, our novel approach treats the Q-learning agent as a spatial regularization actively decoupling and resolving multi-cell overlaps, thus directly demonstrating its validity with a +16.60% absolute jump in accuracy compared to the standard configurations.

**Table 16:** Cross-dataset performance and generalization metrics.

Evaluation dataset	Segmentation accuracy (DSC %)	Classification accuracy (%)
Primary (Kaggle dataset)	95.51	98.00
External (erythrocytesIDB)	90.15	91.40

### External validation and clinical generalizability

To test the robustness of the hybrid framework in different environmental settings (including different staining protocols, variations in illumination artifacts, and different microscope magnifications), the models trained in the main Kaggle repository were applied in zero-shot cross-dataset experiments. For this independent assessment, the public erythrocytesIDB dataset^56^ was employed. As shown in Table 16, a small performance degradation is evident due to large domain shifts and color differences induced by various laboratory staining protocols. Nevertheless, the hybrid instrument possesses robust diagnostic potential, with a classification accuracy of 91.40% on the external set. This indicates that the reinforcement-learning routing layer can adapt to varying shape distributions of overlapping red blood cells on the fly, without retraining hyperparameters over many iterations.

### Technical limitations and failure analysis

Despite the good overall performance, clinical acceptance necessitates an unbiased validation of failure modes. Analysis of misclassifications and rejections turned out two dominant failure scenarios:

- *Extreme clumping and super-imposition* In high-density smears with several sickle cells and normal erythrocytes attainted by clumping of each other in tandem layering (not just simple overlapping), the U-Net segmentation layer sometimes combines boundaries of multiple different cells, making the Q-learning agent miss some key local path nodes.
- *Low contrast and defocusing artifacts* When the microscopic image is strongly affected by lens blur and/or illumination variation, the baseline CNN extraction module extracts low-confidence state features, which results in path routing precision degradation at the localized level. Taking these constraints into account enables the addition of a ‘low-confidence flag’ to future clinical releases to alert laboratory personnel to re-prepare the blood smear slide upon detection of extreme clumping.

### Convergence analysis and algorithmic stability

Since the proposed method employs a reinforcement learning technique (Q-learning) applied to dynamic pixel-coordinate-based state spaces, it is crucial to investigate the system’s numerical stability and convergence behavior to ensure clinical diagnostic reliability. An unpredictably nonconvergent model, or one that wildly oscillates from one training period to the next, certainly cannot be trusted to routinely guide automated microscopy. To assess this stability, the trajectory training loop was executed over 5,000 separate episodes. The training continuum flips through two discrete phases.

- *Exploration and stochastic adaptation (Episode 1 to Episode 1,500):* At the beginning of the training, the *ε * greedy exploration rate is high ($\epsilon \rightarrow 1.0$), which means that the tracking agent has to take a lot of random directional actions to explore the coordinate grid. As a result, the cumulative reward drops drastically due to the continuous path-step penalties (-1) and the boundary-violation penalties (-5). The TD loss is also somewhat noisy at this point, as the Q-table entries are being overwritten rapidly.
- *Exponential policy adaption (Episodes 1501 to 2500)* With the exploration parameter decaying geometrically ($\epsilon \rightarrow 0.1$), a distinct pattern of performance appears. The accumulated reward curve has a very steep, nearly vertical change that quickly moves into positive land. At the same time, the average TD error also decays exponentially and falls below 0.15. This correlated ascent shows that the agent is successfully combining local deep CNN feature vectors at spatial coordinate states with learning to route around normal cells to pinpoint abnormal sickle variants.
- *Exploitation and policy stabilization (Episodes 2501-5000)* With the gradual decay in the exploration parameter comes a shift towards an increasing reliance on the learned action-value policies by the agent. After 2500, the curve of cumulative rewards rises steeply and monotonically until it levels off asymptotically near its mathematical maximum. At the same time, the TD loss decreases monotonically and converges to zero. This striking asymptotic behavior demonstrates that the reward functions designed in Section "Formulation of agent learning for sequential cell tracking" can effectively guide the tracking window along the boundaries of overlapping cells and prevent it from being trapped in infinite loops or flip-flop state oscillations. Such a sharp convergence result mathematically supports the accuracy, consistency, and structural stability of our hybrid RL-CNN system for long-run experiments.

### Statistical significance testing

To thoroughly verify that the performance gains of the proposed Hybrid RL-CNN framework are significant and consistent, we performed the formal statistical significance test. The use of a single maximum-accuracy point hides the variation induced by data partition. Hence, the proposed model was tested with three baselines, Standalone CNN, Decoupled Sequential CNN and YOLOv9+MsR-CNN, on 30 independent, randomized, stratified validation runs.

#### Global evaluation via the Friedman test

Since classification accuracies from multiple training runs are not guaranteed to be uniformly Gaussian distributed, we resorted to the nonparametric Friedman test. This examines the global null hypothesis ($H_0$) that the performance distributions of all four automated pipelines are the same among them. The Friedman rank statistic is computed by Eq. 23.

23 $$\begin{aligned} \chi _r^2 = \frac{12}{N \cdot k(k+1)} \sum _{j=1}^{k} R_j^2 - 3N(k+1) \end{aligned}$$

where *N = 30* the number of independent test runs, *k = 4* is the number of models being tested. and $R_j$ is the sum of the ranks assigned to the model *j* over all runs. The analysis produced a global statistic of $\chi _r^2=54.20$. The associated asymptotic p-value was computed as $p = 1.12 \times 10^{-11}$. Since this value is far below our predetermined threshold for significance (*α *) = 0.05), the null hypothesis is rejected, signifying that there is a significant difference in the performance of the models.

**Table 17:** Pairwise Wilcoxon signed-rank evaluation metrics (proposed vs. baselines).

Comparison pair	Positive ranks sum (*W+*)	Negative ranks sum (*W-*)	Calculated *p*-value
Proposed model vs. standalone CNN	465.0	0.0	$1.73 \times 10^{-6}$
Proposed model vs. decoupled pipeline	438.0	27.0	$4.11 \times 10^{-5}$
Proposed model vs. YOLOv9 + MsR-CNN	402.5	62.5	$2.04 \times 10^{-3}$

#### Post-hoc verification via the wilcoxon signed-rank test

To better isolate the pure statistical effect of our RL-TN layer, we ran the Wilcoxon Signed-Rank test for a post hoc pairwise comparison. This test is a direct assessment of the proposed Hybrid RL-CNN with the Standalone CNN classifier baseline, which is a traditional approach. The accuracy differences ($d_i = x_{\text {Hybrid}, i} - x_{\text {CNN}, i}$) were computed for each of the 30 runs, ranked according to absolute value, and given signed ranks. The Wilcoxon statistics, $W^+$ and $W^-$, are the sums of ranks of positive and negative differences in Eq. 24.

24 $$\begin{aligned} W = \min \left( \sum R_i^+, \sum R_i^-\right) \end{aligned}$$

Table 17 below states the non-parametric parameter results from this pairwise comparison. The comparison between the presented Hybrid RL-CNN model and the traditional standalone CNN yielded a negative rank sum of $W^- = 0.0$, indicating that the architecture formulated using the reinforcement learning method was superior to that of the individual classifier across all test slices. The corresponding two-tailed asymptotic *p*-value ($1.73 \times 10^{-6}$) is well below the usual alpha level of 0.05. This suggests that the framework’s ability to route around intersecting cell outliers offers a statistically significant advantage, hence our performance claims relative to classical ML baselines.

## Model generalizability and multi-center external validation

Although our reinforcement learning scheme exhibits strong performance on our baseline dataset, clinical translation is based on the ability of the model to generalize well across varying institutional acquisition protocols and staining methods. To avoid possible overfitting on center-specific image patterns, work in the future will be aimed at the following three aspects: extensive validation on publicly available benchmark blood smear pools (such as the standard erythrocyte image databases) and blind independent multi-center external cohorts. The inclusion of multi-center validation datasets, collected with different optical magnifications and using different digital microscope hardware, will pose a formidable challenge to the agent’s reward landscape and spatial path regularization and will pave the way to robust performance in varying real-world clinical conditions.

### Computational scalability and practical clinical deployment

Implementing advanced algorithmic methods from research settings to standard clinical operations mandates a thoughtful assessment of computational scalability, inference latency, and hardware limitations. Although training RL agents can be computationally demanding because it requires intensive iterative feedback and policy optimization, our proposed path regularization module for the inference stage is computationally efficient in the deployment stage. Instead of performing dense global re-evaluations on the whole high-resolution WSI, the RL agent keeps the processing overhead low by performing local state space around ambiguous cell boundaries. Scalability is achieved in each of our disparate clinical imaging situations, from high-throughput centralized laboratory digital pathology to limited-resource point-of-care diagnostic devices, by separating the computationally heavy feature extraction from the lightweight sequential policy execution. Concretely, the base U-Net encoder operates on regional feature maps in a single feed-forward pass, and the localized Q-learning agent breaks down multi-cell overlaps with small computational budgets.

### Limitations

Although our framework exhibits a significant increase in performance on the primary validation sets, we would like to explicitly state the limits of what we consider to have been externally validated at this time. The maximum accuracy (98.00%) obtained by the combined Q-learning and CNN framework is specific to imaging modalities with inherent structural distribution properties. Since our RL agent acts on local feature maps produced by the convolutional backbone, a drastic domain shift in the low-level image texture could distort the expected state-space representation ($s_t$), leading to destabilization of the learned policy ($\pi ^*$). Hence, these results cannot be extrapolated to a one-size-fits-all clinical diagnostic tool across all imaging modalities, although successful cross-dataset evaluation is shown in Section "Results and discussion". The real clinical validation would need to be a prospective, multicenter study using raw, unmanipulated blood smears obtained with an array of microscopes representative of the field’s spectrum. Subsequent research will focus on adding unsupervised domain adaptation (UDA) layers to mathematically normalize feature extraction before the RL decision loop, thereby explicitly addressing acquisition-artifact drift. While the hybrid RL-CNN framework proposed displays superior results under the commonly used evaluation criteria, a clear path to clinical translation should include an explicit statement of operational limitations, physical bounds, and technical corner cases. Identification of such constraints gives a realistic perspective on diagnostic safety and outlines potential future work.

- *Extreme three-dimensional cellular superimposition* The main bottleneck in processing is the analysis of very thick blood films with severe cell clumping (multiple erythrocytes vertically stacked or overlapping).
- *Sensitivity to optical focus drift and low contrast* The process can only be as reliable as the physical quality of the digital smear capture.

## Conclusion

In this work, we present a closed-loop hybrid model that compensates for such an error cascade in single-shot models of hematological images when they are subjected to dense and occluded erythrocyte fields. By combining a front-end U-Net segmentation layer with an interactive back-end Q-learning pathfinding agent, the architecture considers the segmented boundaries as a dynamic coordinate grid. Instead of performing global pixel searches, the agent employs a localized tracking window and stepwise reinforcement rewards to separate neighboring cell boundaries. The experimental results from 30 independent stratified cross-validation runs demonstrate the method’s robustness under the same experimental setup. The classification performance of the proposed scheme was 97.84±0.14%, dice similarity coefficient (DSC) was 95.38±0.11%, the precision was 97.62±0.16%, and Recall was 97.45± 0.18%. Global Friedman testing ($\chi _r^2 = 54.20, p < 0.0001$) and post-hoc pairwise Wilcoxon ($W^- = 0.0, p = 1.73 \times 10^{-6}$) validations highlight that these differences are statistically significant. *91.40%* accuracy was achieved by evaluating the model on the erythrocytesIDB corpus as our external validation set, demonstrating the strong generalizability of the proposed work. Yet its full clinical implementation is hindered by technical limitations. The performance degradation caused by extreme three-dimensional cell stacking, optical focus drift, and low-contrast staining artifacts is evident in the failure analysis. Moreover, the offline training phase incurs an exploration-latency penalty prior to policy convergence, but inference is fast and linear in $N _ {cells}$ ($\mathcal {O}(N_{cells})$). Upcoming work will relax these limitations by including multi-Z-plane focal stacking, adaptive continuous reward scaling, and distributed Multi-Agent Reinforcement Learning (MARL).

### Future research directions

The present work provides a solid basis for several lines of future research. First, by extending the architecture to multi-Z-plane focal stacks, the U-Net can resolve overlapping cell membranes in 3D, thereby further mitigating feature corruption in tightly packed regions. Second, the agent’s robustness to staining inconsistencies and optical defocus will be improved by substituting static scalar rewards with gradient-aware adaptive rewards functions, coupled with contrast autotuning. Third, a future direction involves a transition from the single-agent paradigm to a Multi-Agent Reinforcement Learning (MARL) scenario, enabling parallel spatial searching throughout the image matrix, thereby providing a significant reduction in search latency and opening a path for the deployment of the method on edge devices within the constraints of the clinical environment. Future work will consider implementing the hybrid framework on edge-computing laboratory equipment connected to the Internet of Medical Things (IoMT) to bridge the gap between standalone laboratory sample investigation and point-of-care diagnostics. A prime issue in computer-assisted diagnosis is rendering the decisions transparent when dealing with morphological boundaries that straddle statistical gray zones.

## Supplementary Information

Supplementary Information.

## Data Availability

All data supporting this study are openly available in the paper and online^57^ as open-source, without restrictions.

## Code availability

The code used in this study is available at^58^, which includes configuration files, source code, and a user manual.
